# Biphasic Metabolism and Host Interaction of a Chlamydial Symbiont

**DOI:** 10.1128/mSystems.00202-16

**Published:** 2017-05-30

**Authors:** Lena König, Alexander Siegl, Thomas Penz, Susanne Haider, Cecilia Wentrup, Julia Polzin, Evelyne Mann, Stephan Schmitz-Esser, Daryl Domman, Matthias Horn

**Affiliations:** a Department of Microbiology and Ecosystem Science, University of Vienna, Vienna, Austria; b Department for Farm Animal and Public Health in Veterinary Medicine, Institute of Milk Hygiene, Milk Technology and Food Science, University of Veterinary Medicine, Vienna, Austria; University of Illinois at Urbana-Champaign

**Keywords:** *Protochlamydia*, RNA-seq, chlamydia, developmental cycle, gene expression, host-microbe interaction, metabolism, symbiont, type III secretion system

## Abstract

Chlamydiae are obligate intracellular bacteria comprising well-known human pathogens and ubiquitous symbionts of protists, which are characterized by a unique developmental cycle. Here we comprehensively analyzed gene expression dynamics of *Protochlamydia amoebophila* during infection of its *Acanthamoeba* host by RNA sequencing. This revealed a highly dynamic transcriptional landscape, where major transcriptional shifts are conserved among chlamydial symbionts and pathogens. Our data served to propose a time-resolved model for type III protein secretion during the developmental cycle, and we provide evidence for a biphasic metabolism of *P. amoebophila* during infection, which involves energy parasitism and amino acids as the carbon source during initial stages and a postreplicative switch to endogenous glucose-based ATP production. This fits well with major transcriptional changes in the amoeba host, where upregulation of complex sugar breakdown precedes the *P. amoebophila* metabolic switch. The biphasic chlamydial metabolism represents a unique adaptation to exploit eukaryotic host cells, which likely contributed to the evolutionary success of this group of microbes.

**IMPORTANCE** Chlamydiae are known as major bacterial pathogens of humans, causing the ancient disease trachoma, but they are also frequently found in the environment where they infect ubiquitous protists such as amoebae. All known chlamydiae require a eukaryotic host cell to thrive. Using the environmental chlamydia *Protochlamydia amoebophila* within its natural host, *Acanthamoeba castellanii*, we investigated gene expression dynamics *in vivo* and throughout the complete chlamydial developmental cycle for the first time. This allowed us to infer how a major virulence mechanism, the type III secretion system, is regulated and employed, and we show that the physiology of chlamydiae undergoes a complete shift regarding carbon metabolism and energy generation. This study provides comprehensive insights into the infection strategy of chlamydiae and reveals a unique adaptation to life within a eukaryotic host cell.

## Abstract (precis)

Chlamydiae are known as major bacterial pathogens of humans, causing the ancient disease trachoma, but they are also frequently found in the environment where they infect ubiquitous protists such as amoebae. All known chlamydiae require a eukaryotic host cell to thrive. Using the environmental chlamydia *Protochlamydia amoebophila* within its natural host, *Acanthamoeba castellanii*, we investigated gene expression dynamics *in vivo* and throughout the complete chlamydial developmental cycle for the first time. This allowed us to infer how a major virulence mechanism, the type III secretion system, is regulated and employed, and we show that the physiology of chlamydiae undergoes a complete shift regarding carbon metabolism and energy generation. This study provides comprehensive insights into the infection strategy of chlamydiae and reveals a unique adaptation to life within a eukaryotic host cell.

## INTRODUCTION

Chlamydiae represent an ancient group of obligate intracellular bacteria (1). Hundreds of millions of years of evolution in association with eukaryotic host cells gave rise to successful pathogens of humans and diverse, globally distributed environmental chlamydiae associated with protists and animals (2–4). The human pathogen *Chlamydia trachomatis* (family *Chlamydiaceae*) is a frequent cause of sexually transmitted diseases and sight-threatening infections, affecting more than 180 million people each year (5, 6). While the chlamydiae are currently represented by a limited number of families, molecular evidence suggests the existence of a tremendous diversity in diverse habitats (>250 unexplored families), including potential new emerging pathogens (3, 7–11). Despite this diversity, all chlamydiae undergo a biphasic developmental cycle (2, 12). A detailed knowledge of this defining feature is the key to understanding the biology, evolution, and host interactions of this unique group of microbes.

The chlamydial developmental cycle consists of two morphologically and physiologically distinct stages termed elementary bodies (EBs) and reticulate bodies (RBs) (2, 12). Infection of eukaryotic host cells occurs at the EB stage. After host cell entry, EBs differentiate quickly into replicating RBs, which reside within a cytoplasmic vacuole called an inclusion, and undergo several rounds of cell division. RBs asynchronously convert back to EBs, which are subsequently released into the extracellular environment by host cell lysis or nonlytic extrusion (12, 13). The developmental cycle has mainly been studied for chlamydial pathogens, such as *C. trachomatis* and *C. pneumoniae*. Although there are slight variations between organisms, the cycle is well conserved among all known chlamydiae and likely has evolved before their diversification (1, 14–18).

DNA microarray and quantitative PCR studies have been instrumental in elucidating the molecular basis of the developmental cycle of *C. trachomatis* and *C. pneumoniae*, which found that infection and differentiation processes are accompanied by marked temporal changes of gene expression (19–24). The recent sequencing of entire transcriptomes (RNA-seq) using next-generation sequencing platforms improved the detection of early chlamydial genes (25), facilitated the identification of transcription start sites, and led to the discovery of novel transcripts (26, 27). However, this powerful technology has not yet been applied to study the chlamydial developmental cycle over the entire course of infection. In addition, although this method has the potential to interrogate microbe and host transcriptomes simultaneously (28), technical challenges have hampered its application for the analysis of chlamydiae, with only a single study available so far (25).

*Protochlamydia amoebophila* (hereafter referred to as *Protochlamydia*; a member of the family *Parachlamydiaceae*) is a well-studied symbiont (29–31). Of key importance, *Protochlamydia* in its natural *Acanthamoeba* host represents an *in vivo* infection model to study the dynamics of host-microbe interactions, and the model does not rely on the use of immortalized cell lines. Studying these environmental counterparts of the *Chlamydiaceae* has led to a number of discoveries with important implications for our understanding of the basic biology of all chlamydiae, including the discovery of peptidoglycan and metabolic activity of EBs (18, 32–37). Yet, a detailed analysis of the *Protochlamydia* developmental cycle and the underlying gene expression dynamics has thus far been strikingly lacking.

In this study, we describe the course of the infection and investigate the transcriptome of *Protochlamydia* and its amoeba host at key time points by RNA-seq. Highlighting conserved chlamydial and unique protochlamydial developmental events we aimed at understanding general chlamydial biology, including the role of protein secretion and the metabolic strategy accompanying the transitions between developmental stages. Our analyses revealed the conservation of a pronounced temporal gene expression profile during the developmental cycle among amoeba-associated chlamydiae and chlamydial pathogens and support a model in which a major shift in the metabolism of *Protochlamydia*—from energy parasitism to endogenous energy production—occurs during differentiation.

## RESULTS AND DISCUSSION

### Three main temporal classes of gene expression.

**FIG 1:**
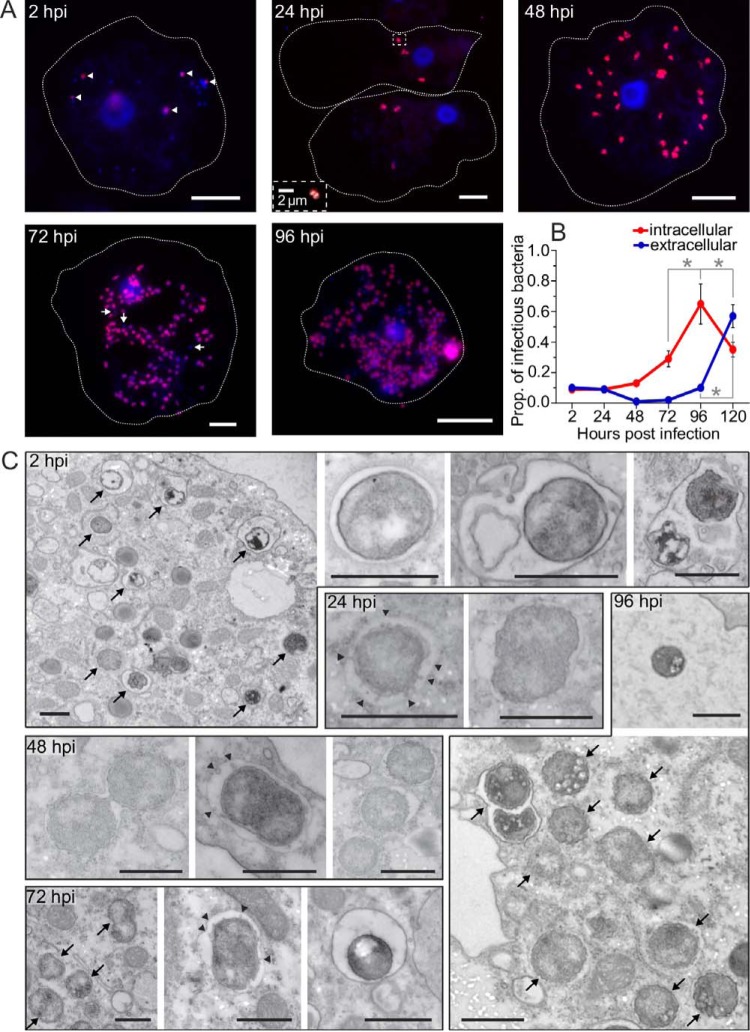
Developmental cycle of *Protochlamydia amoebophila*. (A) Fluorescence *in situ* hybridization in combination with DAPI staining was used to differentiate between RBs (pink) and EBs (blue). At 2 h postinfection EBs clearly dominate, but a few cells have already started to convert to RBs (white arrowheads). Exclusively RBs were detected at 48 hpi. The first EBs (white arrows) were seen at 72 hpi. The inset for the 24 hpi image is an enlargement of dividing RBs (to enhance clarity, the DAPI signal is shown in white). Dotted white lines indicate the outlines of amoeba host cells. If not indicated otherwise, all bars represent 10 µm. (B) The course of EB production and release was quantified by collecting intra- and extracellular bacteria, respectively, at indicated time points, and subsequent reinfection of fresh amoebae. The first intracellular EBs were present at 72 hpi, the first release of EBs was observed at 96 hpi. Values that are significantly different (*P* < 0.05) at the various time points by one-way analysis of variance (ANOVA) and Tukey’s posttest are indicated by an asterisk (*n* = 3). Prop., proportion. (C) Transmission electron micrographs visualizing developmental events at the ultrastructural level. Black arrows point to bacterial cells in overview images. Black arrowheads indicate vesicles that were observed in the inclusion lumen from 24 hpi on. Bars = 1 µm.

We investigated the developmental cycle of the amoeba endosymbiont *Protochlamydia* by monitoring the course of infection using fluorescence *in situ* hybridization, 4′,6′-diamidino-2-phenylindole (DAPI) staining, and transmission electron microscopy, and by quantifying the production of infectious EBs (Fig. 1). In summary, *Protochlamydia* in its *Acanthamoeba* host exhibits the characteristic chlamydial developmental cycle (see Text S1  in the supplemental material for details). Completion of the cycle takes 96 h, slightly longer than what has been reported for chlamydiae infecting humans and animals (20, 21). It is, however, shorter than the 6 to 15 days observed for other chlamydiae (14, 17, 38).

10.1128/mSystems.00202-16.1 TEXT S1  Supplemental methods, results, and discussion. Download TEXT S1, PDF file, 0.1 MB. Copyright © 2017 König et al. 2017 König et al. This content is distributed under the terms of the Creative Commons Attribution 4.0 International license.

**FIG 2:**
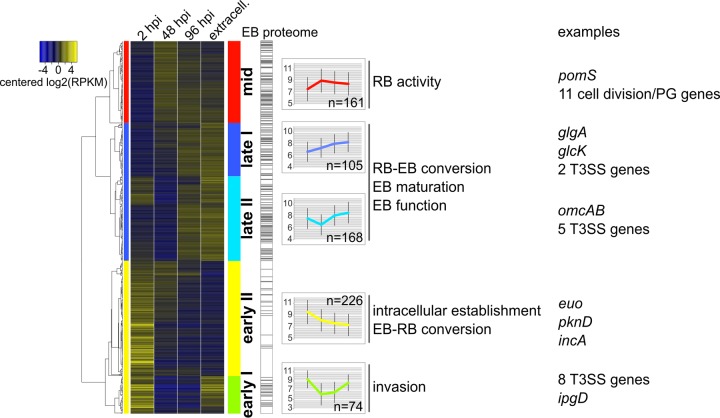
Temporal classes of gene expression during the *Protochlamydia* developmental cycle. A total of 797 genes were detected as differentially expressed; tRNA genes (20 genes), rRNA genes (2 genes), and genes detected only in a single replicate (38 genes) were excluded from further analysis. Clustering identified three main temporal classes of gene sets (colored bars to the left of the heatmap) that could be further divided into five large subclasses (colored bars to the right of the heatmap). The largest group of genes was most highly expressed early (*n* = 304), whereas the expression of the smallest group of genes peaked at midcycle when only RBs were present (*n* = 161). The third main gene cluster generally showed highest expression at the end of the cycle and the extracellular stage (*n* = 273; see Data Set S1 in the supplemental material). Gene products detected in the EB proteome in a previous study (*n* = 231) (39) are indicated in the bar plot next to the heatmap. To illustrate the course of gene expression for each subcluster, the expression values (log_2_ RPKM plotted on the *y* axis) were averaged per time point (*x* axis) and visualized as line plots (error bars indicate standard deviations). Selected gene names are shown for each of the temporal clusters. RPKM, reads per kilobase per million; hpi, h postinfection; extracell., extracellular; PG, peptidoglycan synthesis; T3SS, type III secretion system.

For RNA sequencing, we selected four time points that mark crucial developmental events during infection: 2 h postinfection (hpi) representing the start of EB-to-RB transition, 48 hpi as the peak of RB activity, 96 hpi covering secondary differentiation, and released EBs (Fig. 1 and Text S1). Despite different sequencing read numbers that could be mapped to the *Protochlamydia* genome at each time point (Table S1), the sequencing depths of all samples and the similarities between biological replicates were sufficient (Fig. S1 and Text S1) to identify 737 protein-coding genes that were differentially expressed (DE) at least once during the developmental cycle (corresponding to 51% of all genes with expression data) (Fig. 2). All other expressed genes were considered constitutively expressed genes (Fig. S2A). Hierarchical clustering of DE genes by expression profiles revealed three main temporal classes of gene expression (Fig. 2 and Data Set S1), whose expression profiles are broadly reflected in the EB proteome determined previously (39) (Fig. 2). Importantly, these three main temporal groups (termed early, mid, and late genes) correspond well with those found in microarray studies of *Chlamydiaceae* (23). Therefore, the pronounced transcriptional switches accompanying EB-to-RB transition and intracellular establishment, RB activity, and RB-to-EB transition are conserved among amoeba-associated chlamydiae and the *Chlamydiaceae* and represent a hallmark of all chlamydiae. However, given the proportionally larger number of differentially regulated genes upon entry and early developmental events we found for *Protochlamydia* (Fig. S3 and Text S1), amoeba-associated chlamydiae require a higher degree of adjustment when transitioning to the intracellular environment.

10.1128/mSystems.00202-16.2 FIG S1  Similarity of *Protochlamydia* gene expression values between replicates and time points. The Euclidean distances between all normalized expression values (reads per kilobase per million [RPKM]) of each replicate and time point are shown, with darker colors meaning high similarity. The order of the samples is based on hierarchical cluster analysis of the distances using the complete linkage method—the clustering being illustrated by the dendrograms. The samples cluster by time point and therefore correctly reflect the experimental design. For subsequent analyses, genes were considered to be expressed at a particular time point when we obtained expression values in two or three replicates. The contribution of genes for which expression was detected in only two replicates is indicated in the right panel. Relatively low sequencing coverage at 2 h postinfection (hpi) explains the higher numbers of these genes at this early time point, and consequently also the larger distance between the 2 hpi replicates. Excl., excluding. Download FIG S1, PDF file, 0.2 MB. Copyright © 2017 König et al. 2017 König et al. This content is distributed under the terms of the Creative Commons Attribution 4.0 International license.

10.1128/mSystems.00202-16.3 FIG S2  (A) Gene expression heatmaps of constitutively expressed genes and (B) genes involved in peptidoglycan synthesis and cell division. (A) All genes that were detected to be expressed (in at least two replicates) but were found not to be significantly differentially expressed (*n* = 712) were termed constitutively expressed. Average gene-wise expression values (log_2_ reads per kilobase per million [RPKM]) were hierarchically clustered based on Euclidean distances using the complete linkage method. Five clusters representing five different expression levels were defined on the basis of the dendrogram’s branching pattern. Accordingly, we found 9 very highly expressed genes, 60 highly expressed genes, 131 genes with medium expression strength, 328 genes with medium-low expression strength, and 184 genes with low expression. Each cluster was tested for the presence of overrepresented COG classes and clusters, KEGG pathways, and GO terms for which only categories with a false discovery rate (FDR) below 0.05 are listed. In addition, a few selected genes are shown. Corresponding proteins that were detected in *P. amoebophila* EBs in a previous study (B. S. Sixt, C. Heinz, P. Pichler, E. Heinz, J. Montanaro, et al., Proteomics 11:1868–1892, 2011, https://doi.org/10.1002/pmic.201000510) are indicated by black lines (*n* = 234). (B) Average expression values (log_2_ RPKM) for genes involved in peptidoglycan synthesis and cell division (N. Jacquier, P. H. Viollier, and G. Greub, FEMS Microbiol Rev 39:262–275, 2015, https://doi.org/10.1093/femsre/fuv001) were hierarchically clustered (average linkage) based on Pearson correlation distances. Genes found to be differentially expressed between any two consecutive time points are marked with an asterisk. hpi, h postinfection; extracell., extracellular. Download FIG S2, PDF file, 0.7 MB. Copyright © 2017 König et al. 2017 König et al. This content is distributed under the terms of the Creative Commons Attribution 4.0 International license.

10.1128/mSystems.00202-16.4 FIG S3  Gene expression dynamics during the *Protochlamydia* developmental cycle. The intersecting gene sets of statistically significantly up- or downregulated genes between successive time point pairs are shown. Each color represents a different set of differentially expressed (DE) genes, with the height of the bar indicating the number of genes. For example, at 2 h postinfection (hpi), there are a total of 250 significantly upregulated genes of which 153 genes are downregulated at the next time point (orange bars). Note that there are overlaps between gene sets of two consecutive time points. For instance, from the orange gene set (upregulated at 2 hpi and downregulated at 48 hpi), 22 genes were subsequently also downregulated at 96 hpi (pink boxes). White areas represent DE genes that were detected as significantly regulated only once during the cycle. Download FIG S3, PDF file, 0.2 MB. Copyright © 2017 König et al. 2017 König et al. This content is distributed under the terms of the Creative Commons Attribution 4.0 International license.

10.1128/mSystems.00202-16.8 TABLE S1  Sequencing and read mapping statistics. Download TABLE S1, PDF file, 0.1 MB. Copyright © 2017 König et al. 2017 König et al. This content is distributed under the terms of the Creative Commons Attribution 4.0 International license.

10.1128/mSystems.00202-16.10 DATA SET S1  Gene expression values and annotation of *Protochlamydia* and *A. castellanii*. Download DATA SET S1, XLSX file, 1.8 MB. Copyright © 2017 König et al. 2017 König et al. This content is distributed under the terms of the Creative Commons Attribution 4.0 International license.

### Temporal partitioning of biological processes during development.

The three main temporal gene expression classes can be further subdivided into five distinct gene expression patterns termed here early I, early II, mid, late I, and late II (Fig. 2). The analysis of these gene sets provides detailed insights into the course of biological processes during chlamydial development.

**FIG 3:**
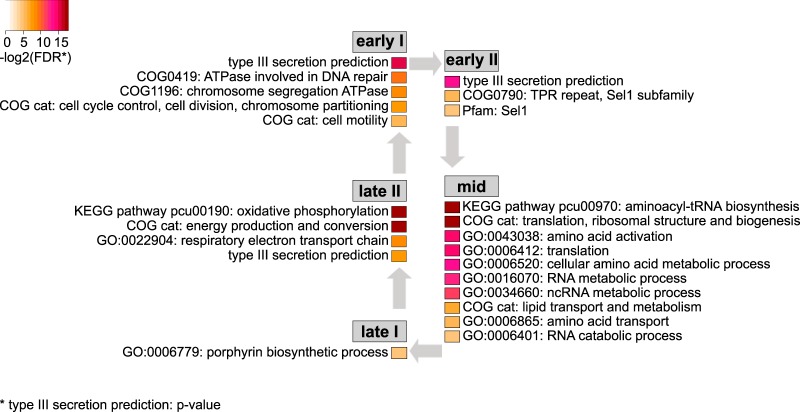
Enrichment of functional categories by temporal class. The overrepresentation of functional categories among genes assigned to temporal classes provides evidence for stage-specific activities during the *Protochlamydia* developmental cycle. Only functional categories that were significantly enriched with a false-discovery rate (FDR) of ≤0.05 are shown here; the color code indicates the degree of significance (dark red indicates highly significant). The significant enrichment of putatively type III secreted gene products was tested using Fisher’s exact test, and only *P* values of ≤0.05 are shown. cat, category.

Early I genes (*n* = 74) are highly expressed at 2 hpi and then strongly downregulated at midcycle. They remain at a lower expression level until late in the development, but transcripts are notably abundant in extracellular EBs (Fig. 2). Some of the genes referred to as “tardy” genes in a previous *C. pneumoniae* microarray study (20) follow a similar expression pattern, yet this transcriptional profile is strikingly pronounced in *Protochlamydia*. mRNA has been detected in EBs (20, 22, 26, 27, 40), which would be consistent with transcriptional activity, but EBs are generally considered transcriptionally silent (23). mRNA detected in EBs could thus represent “carryover” mRNA (19), for which it has been unclear whether it is actually used as the templates for translation (23). Here we propose that these transcripts are preserved in EBs to be readily available and functional during initial infection events. Consistent with this view, we find many genes encoding structural type III secretion proteins in this temporal class, as well as a number of predicted type III effectors (Fig. 2 and 3). Among those is a *Protochlamydia*-specific orthologue of the major enterobacterial virulence factor IpgD, which in *Shigella* facilitates host cell entry (41) and might be functionally similar to the *Chlamydiaceae* Tarp (42, 43). Taken together, the conspicuous expression pattern of the early I gene set points at a function in invasion of the amoeba host.

A second set of early genes termed early II (*n* = 226) is upregulated at 2 hpi, subsequently downregulated, and—in contrast to early I genes—remains at a lower expression level for the rest of the cycle, including the EB stage (Fig. 2). These genes thus likely function mainly in EB-to-RB conversion and are important for establishing the intracellular niche. Consistently, this temporal class contains genes that in *C. trachomatis* follow a similar expression pattern (e.g., the chlamydia-specific inclusion membrane proteins and the transcriptional repressor of known late genes Euo) (19, 22, 23). This temporal class, however, was dominated in *Protochlamydia* by a large number of putative eukaryotic-like effectors with many exhibiting Sel1-like repeat domains or belonging to the recently identified large gene families PEX1 and PEX2 encoding proteins potentially involved in modification of host ubiquitination processes (Fig. 3 and Fig. S4) (33). Together, this indicates that while conserved chlamydial processes are at work during early niche establishment and RB-to-EB differentiation, *Protochlamydia* also employs a set of species-specific effector proteins during this stage.

The midcycle gene set comprises genes sharply upregulated at 48 hpi, when mainly RBs are present (Fig. 1). Transcripts for many of these genes were also detected at later stages at similar expression levels (Fig. 2) when the developmental cycle becomes more asynchronous. At this peak of RB activity, cellular processes like translation and metabolic activity (lipid and amino acid transport and metabolism) are most prominent (Fig. 3). Consistent with high metabolic activity, a number of genes responsible for cell division and peptidoglycan synthesis were upregulated, or by trend increased at midcycle (Fig. S2B). Analyzing the distribution of conserved and species-specific genes across the observed temporal gene expression classes revealed that the midcycle gene set was dominated by genes present in all chlamydiae, thereby confirming the role of chlamydial core genes in RB metabolism and proliferation (Fig. S5 and Text S1).

We found two subsets of late genes that are both characterized by an increased expression at the end of the cycle at 96 hpi and by generally high transcript levels at the EB stage (Fig. 2). Expression of late I genes (*n* = 105) already begins to increase at midcycle; the upregulation of late II genes (*n* = 168) is slightly delayed. These genes are thus involved in RB-to-EB transition, EB function, and EB maintenance.

Late I genes are enriched in genes involved in biosynthesis of porphyrins such as the cofactor heme required for the production of cytochromes (Fig. 3). This temporal class also includes genes involved in glucose and glycogen metabolism, such as glucokinase (*glcK*) and glycogen synthase (*glgA*), suggesting that glucose-based energy production is important at this stage. This trend is supported by the functions of late II genes, which are enriched in genes responsible for energy generation, including the electron transport chain for ATP synthesis (Fig. 3). Consistent with the presence of type III secretion systems on chlamydial EBs, we find many genes encoding structural proteins and known (CopB) and predicted effectors among the late I and late II gene sets (Fig. 3) and (Fig. S4).

### A temporal model for chlamydial type III secretion.

The type III secretion system is an evolutionarily well conserved major virulence factor that was present in the last common ancestor of all known chlamydiae (1, 44, 45). Here we observed that genes involved in type III secretion, including structural components, chaperones, and effectors, belong to all five temporal gene sets and show strikingly different expression profiles (Fig. S4). While the basal apparatus and most components of the C-ring complex required for structural integrity and secretion activity (e.g., *sctD*, *sctC*) are expressed mid to late during RB/EB transition, notably lacking at this stage is a pronounced expression of the gene encoding the needle protein SctF. In fact, the transcript levels of the needle protein, the translocon CopB, most known chaperones, most putative effectors, as well as additional inner membrane components and the ATPase increase only later during maturation of EBs but then continue to be highly expressed early during infection (Fig. S4).

10.1128/mSystems.00202-16.5 FIG S4  Expression patterns of genes encoding components of the type III secretion system and its (putative) effectors. All genes that met the following criteria were included: (i) genes that have been previously reported to encode components involved in *Protochlamydia* type III secretion (A. Collingro, P. Tischler, T. Weinmaier, T. Penz, E. Heinz, et al., Mol Biol Evol 28:3253–3270, 2011, https://doi.org/10.1093/molbev/msr161); (ii) genes that are orthologues of putative *C. trachomatis* effector proteins or structural components and chaperones of the chlamydial type III secretion system (H. J. Betts-Hampikian and K. A. Fields, Front Microbiol 1:114, 2010, https://doi.org/10.3389/fmicb.2010.00114); (iii) genes that were annotated to contain eukaryotic-like domains such as ankyrin repeats (ANK), Sel1-like repeats (Sel1), F-boxes (F-box), leucine-rich repeats (LRR, PEX1), tetratricopeptide repeats (TPR, PEX2), BTB/POZ domains (PEX1), and GDP-GTP exchange domains (RasGEF, RhoGEF); (iv) genes that were predicted to encode inclusion membrane proteins (put. inc) (E. Heinz, D. D. Rockey, J. Montanaro, K. Aistleitner, M. Wagner, et al., J Bacteriol 192:5093–5102, 2010, https://doi.org/10.1128/JB.00605-10); and (v) genes that encode putative effectors based on expression pattern and genomic location (data not shown). Differentially expressed genes are marked with an asterisk. Temporal classes are indicated by colored bars. The sct unified nomenclature for the naming of type III secretion system genes was used (C. J. Hueck, Microbiol Mol Biol Rev 62:379-433, 1998). RPKM, reads per kilobase per million; hpi, h postinfection; extracell., extracellular; PEX1, *Protochlamydia* expanded gene family 1; PEX2, *Protochlamydia* expanded gene family 2 (D. Domman, A. Collingro, I. Lagkouvardos, L. Gehre, T. Weinmaier, et al., Mol Biol Evol 31:2890–2904, 2014, https://doi.org/10.1093/molbev/msu227); put, putative; Ctr, *C. trachomatis*. Download FIG S4, PDF file, 1.3 MB. Copyright © 2017 König et al. 2017 König et al. This content is distributed under the terms of the Creative Commons Attribution 4.0 International license.

10.1128/mSystems.00202-16.6 FIG S5  Expression of species-specific and chlamydial core genes. (A) Heatmap showing the distribution of temporarily regulated *Protochlamydia* genes at different levels of taxonomic conservation. The color indicates the percentage of differentially expressed (DE) genes per temporal class and taxonomic group. The absolute numbers of DE genes per temporal class and taxonomic group are indicated; the total numbers of DE genes per taxonomic group are shown in brackets. Note that the early gene set is dominated by species-specific genes (32%), whereas chlamydial core genes are most abundant at midcycle (44%). (B) Nearly all chlamydial core genes are expressed in *Protochlamydia*, with constitutively expressed genes contributing 62%. Download FIG S5, PDF file, 0.2 MB. Copyright © 2017 König et al. 2017 König et al. This content is distributed under the terms of the Creative Commons Attribution 4.0 International license.

**FIG 4:**
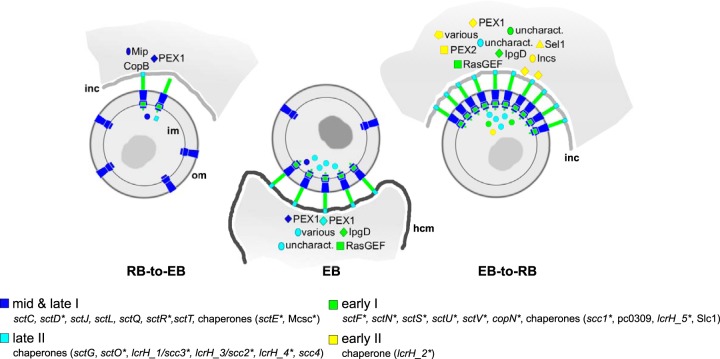
Course of *Protochlamydia* type III secretion system activity during the developmental cycle. This model is based on the observation that structural components of the type III secretion system and its (putative) effectors are expressed at different time points during the developmental cycle (Fig. S4). This suggests a scenario in which novel, fully assembled, and thus functional secretion systems occur only late in the developmental cycle, and type III secretion reaches its full capacity and highest activity during early stages of the infection. The indicated polarity of the active type III secretion system has been shown for *C. trachomatis* (54), but it is unclear whether this is also true for *P. amoebophila*, as the symbionts reside within single-cell inclusions. The color code for type III secretion components and effectors (nomenclature according to Hueck [105]) refers to the respective temporal gene expression classes (Fig. 2 and Fig. S4). Circles inside the cells represent chaperones. Differentially expressed components/effectors are labeled with asterisks; PEX1 and PEX2 refer to members of the expanded effector gene families in *Protochlamydia* (33); pc0309 is an ortholog of the putative chaperone encoded by CT274 (106). inc, inclusion membrane; hcm, host cell membrane; im, inner membrane; om, outer membrane.

Our gene expression-centric view of type III secretion thus suggests a stepwise assembly of the *Protochlamydia* type III secretion apparatus, which is consistent with knowledge about type III secretion system assembly in other bacteria (46) and models for *Chlamydiaceae* proposed earlier (45). Our time-resolved model for the role of type III secretion during the developmental cycle includes three major steps (Fig. 4). The first step is the formation of the main parts of novel type III secretion machineries mainly during RB-to-EB differentiation; because of the lack of a pronounced expression of the needle protein and other factors prior to this stage, we propose that type III secretion machineries are mostly incomplete at midcycle and therefore, secretion is less prominent when RBs likely devote most of their resources to proliferation (Fig. 3). At the tail end of RB-to-EB differentiation, SctF is upregulated, and most secretion machineries are then fully equipped. Thus, the second step is marked by functional type III secretion systems that are present on extracellular EBs and can be employed for host cell invasion. The third step is characterized by the peak of type III secretion activity occurring in early RBs, where we observed all structural components, including the needle protein to be continuously highly expressed. Importantly, the major role of type III secretion during early development is also well supported by the largest number of (putative) effectors upregulated at this stage (Fig. 4 and Fig. S4).

It is well-known that type III secretion genes of bacterial pathogens are temporally regulated (47). Moreover, our model is consistent with gene expression data for the *Chlamydiaceae*, which indicate that the large majority of type III secretion component genes are temporally regulated and increasingly expressed from midcycle toward the end (19–21, 48, 49). The model is also supported by proteomic analyses of *Protochlamydia* and *C. trachomatis* EBs and RBs in which most type III secretion system components were identified on prereplicative RBs (50) and EBs (39, 51). The model is also generally in agreement with recent (cryo-)electron tomography analyses of *Protochlamydia and C. trachomatis*, demonstrating the presence of type III secretion systems on host-free EBs (52–54) and late RBs (55). Finally, our interpretation that type III secretion systems are more abundant on early RBs compared to EBs matches electron microscopy data for *Chlamydia psittaci* (56). The model is, however, not consistent with the apparent absence of the type III secretion machinery on *C. trachomatis* EBs noted immediately after host entry in a cryo-electron tomography-based study (54), which would imply that type III secretion is not required at this stage. The latter scenario is difficult to reconcile with the general notion that effector secretion is essential during early development (45, 57). We thus propose that type III secretion is active to facilitate invasion by EBs, but based on the pronounced early gene expression of type III secretion components and effectors—observed here for the first time—is substantially enhanced during establishment of the replicative niche by early RBs (Fig. 4).

### Biphasic metabolism of *Protochlamydia*.

A number of previous observations provide evidence for developmental stage-specific differences of the chlamydial metabolism (reviewed in reference 58). Our comprehensive transcriptomic data set allows us to dissect the activity of individual pathways and metabolic modules during the *Protochlamydia* developmental cycle. As a result, we are able to propose a detailed model for the modulation of chlamydial physiology during infection and extracellular survival.

### Stage-specific energy sources.

**FIG 5:**
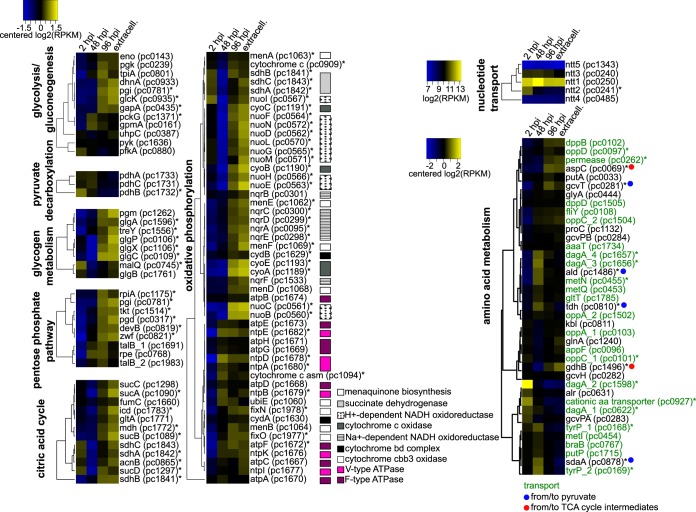
Expression maps of selected metabolic pathways of *Protochlamydia*. A pronounced expression of the ATP/ADP translocase (*ntt1*) at midcycle and an early-to-mid activity of genes involved in amino acid breakdown to pyruvate is observed, whereas pathways involved in central carbon metabolism and energy generation were generally only upregulated at later stages (with the ATPases indicated by purple boxes being notable exceptions). This suggests that a major metabolic shift occurs during the developmental cycle and provides evidence for a stage-specific metabolism. All genes marked with an asterisk were detected to be significantly differentially expressed. RPKM, reads per kilobase per million; hpi, h postinfection; extracell., extracellular.

Chlamydiae are well-known energy parasites facilitated by nucleotide transport proteins, which import host-derived ATP in exchange for ADP (59, 60). Yet, they also harbor the genetic potential to synthesize ATP via oxidative phosphorylation. Our gene expression analysis, however, revealed that in *Protochlamydia*, genes involved in carbon and energy metabolism show a low transcription level at midcycle and are induced only late during development (Fig. 5). At the replicative stage, the import of host-derived ATP is thus likely the most important energy source. The ATP/ADP translocase (*ntt1*) is constitutively expressed at an exceptionally high level (among the top 1% of all expressed genes), and although it was not detected as significantly differentially expressed, its transcription profile clearly peaks at midcycle (Fig. 5). The same trend has been observed for *C. trachomatis* and *C. pneumoniae* (19, 20), and nucleotide transport proteins were most abundant in *C. trachomatis* RBs compared to EBs (51). In addition, ATP but not glucose-6-phosphate stimulated *de novo* protein synthesis in *C. trachomatis* RBs in a chemically defined axenic medium (61).

Because late genes were enriched in respiratory chain genes (Fig. 3), we compiled detailed gene expression heatmaps for all genes known to be part of the chlamydial carbon and energy metabolism pathways (Fig. 5). Strikingly, we also found genes involved in glycolysis, the pentose phosphate pathway, and the tricarboxylic acid (TCA) cycle to be generally upregulated only late during development. In agreement, we observed a pronounced upregulation of genes responsible for glutamate and aspartate breakdown (*gdhB*, glutamate dehydrogenase; *aspC*, aspartate aminotransferase; Fig. 5), whose end products could fuel the TCA cycle.

A notable exception are the V-type and the F-type ATPases whose expression is induced at midcycle, a trend which was also seen for the respective *C. trachomatis* proteins (51). In the absence of other main components of the respiratory chain at this stage, the ATPases likely engage in hydrolysis of ATP (rather than ATP synthesis) and thus act as proton/sodium pumps maintaining the membrane potential—a function well-known for both types of ATPases and proposed for the ATPase in *C. psittaci* RBs (62–64). In *Protochlamydia*, the ATPases continue to be expressed at later stages, when all components of the oxidative phosphorylation pathway are in place. The proton/sodium gradient can then be generated by the other complexes of the respiratory chain, and the ATPases would function in ATP synthesis.

This model fits well with findings for *C. trachomatis*, where proteins involved in the endogenous energy metabolism were detected only in EBs, not in RBs (65), or were found to be more abundant in EBs (51). Moreover, glucose 6-phosphate but not ATP stimulated *de novo* protein synthesis in host-free *C. trachomatis* EBs, and ATP generation was observed at this stage (61). Furthermore, we have previously shown that *C. trachomatis* and *Protochlamydia* EBs require glucose 6-phosphate or glucose, respectively, for maintenance of infectivity during extracellular survival (35). In conclusion, the gene expression data presented here provide compelling evidence that a stage-specific energy metabolism indeed occurs *in vivo* and that it is well conserved among known chlamydiae.

### Amino acids and pyruvate as main carbon source during replication.

**FIG 6:**
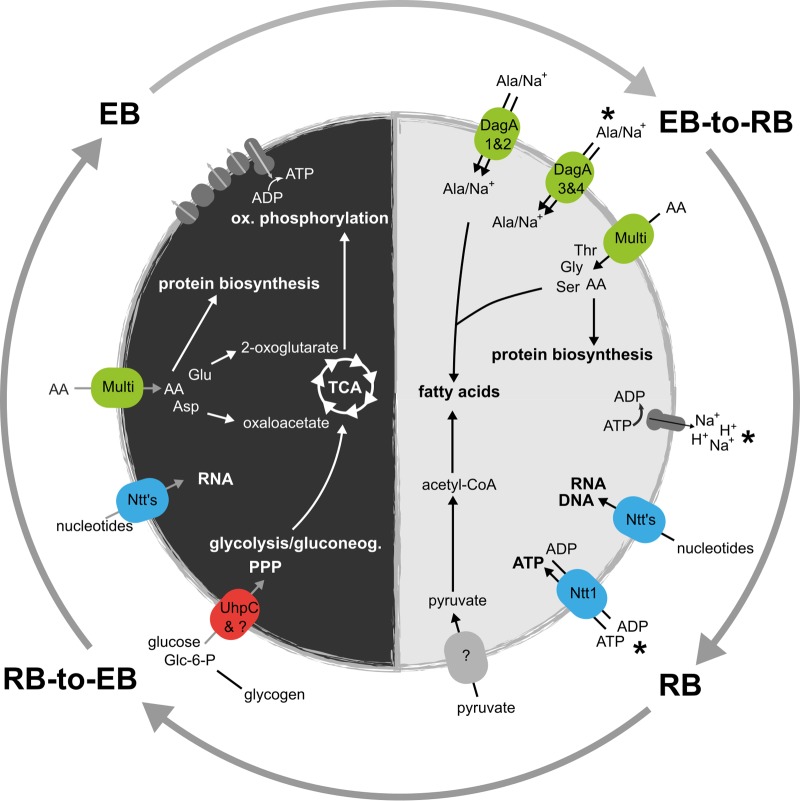
Biphasic metabolism of *Protochlamydia* during development in *Acanthamoeba castellanii*. This model is based on observed transcriptional patterns, enriched functional categories at different developmental stages (Fig. 2, 3, and 5 and Fig. S2A), and independent experimental evidence reported previously (see text for references). Activity of metabolic pathways as inferred from gene expression levels followed similar trends early and at midcycle, and at the two later stages, respectively. This suggests that ATP import and an amino acid-based anabolism prevails during the EB-to-RB transition and RB replication. Later stages are characterized by a glucose-based metabolism and a pronounced increase in the activity of the tricarboxylic acid (TCA) cycle and oxidative (ox.) phosphorylation pathway. Nucleotide transporters (Ntt’s) are shown in blue, and amino acid (AA) and oligopeptide transporters are shown in green. “Multi” indicates that multiple amino acid/peptide transporters with different substrate specificities are expressed. Question marks refer to hypothetical transporters not yet identified. Asterisks indicate an increased expression at the RB stage compared to the early time point. “RNA” denotes transcription, whereas “DNA” indicates DNA replication. Glc-6-P, glucose 6-phosphate; PPP, pentose phosphate pathway; glyconeog., gluconeogenesis.

Another consequence of the observed expression of genes involved in breakdown of glucose predominantly at the postreplicative stage is that glucose cannot represent the major carbon source at the RB stage. In fact, chlamydiae acquire the majority of cell building blocks such as amino acids, nucleotides, and certain lipids from the host cell (66). Transport proteins required for the uptake of those compounds exist, and most of the 24 known amino acid transporters of *Protochlamydia* are expressed early and midcycle, providing all the substrates required for protein synthesis in RBs (Fig. 5). The importance of host-derived amino acids for chlamydial protein synthesis is well supported by a recent isotopologue profiling study of *C. trachomatis* (67). While no external carbon source might thus be required for protein, DNA, and RNA synthesis, chlamydiae clearly need carbon to synthesize branched-chain fatty acids for the generation of phospholipids (68). Consistent with this notion, lipid metabolism was induced at the replicative stage in *Protochlamydia* (Fig. 3). This includes the pyruvate dehydrogenase genes (*pdhABC*), which were upregulated early and at midcycle and catalyze the conversion of pyruvate to acetyl coenzyme A (acetyl-CoA), the precursor of fatty acid biosynthesis (Fig. 5). The pyruvate required for this step could be imported directly from the host as suggested for *C. psittaci* (69), although no pyruvate transporter has yet been identified in *Protochlamydia* or *Chlamydiaceae*. Pyruvate cannot be generated from host-derived nucleotides or host lipids, as *Protochlamydia* lacks the genetic repertoire for breakdown of those compounds. However, genes encoding components involved in degradation of amino acids are present and were indeed most highly expressed early (*sdaA*, l-serine dehydratase) and at midcycle (*tdh*, l-threonine 3-dehydrogenase; *ald*, alanine dehydrogenase; *gcvT*, T protein of the glycine cleavage complex; Fig. 5). These enzymes catabolize alanine, threonine, glycine, and serine to pyruvate, which may then serve as the substrate for branched-chain fatty acid synthesis. The increased number of gene copies encoding a specific transporter for alanine (*dagA*) and their early and midcycle expression further support a model in which amino acids provide the carbon required for fatty acid biosynthesis in *Protochlamydia* RBs (Fig. 6).

### Metabolism of EBs.

Many of the late genes involved in carbon and energy metabolism are still highly expressed at the EB stage in *Protochlamydia*. Remarkably, this is also the case for genes involved in transcription and protein synthesis. This includes the RNA polymerase, amino acid transporters, and many translation-related genes (Fig. 5 and Fig. S2A). Our transcriptome data thus support a number of early observations and recent findings suggesting that chlamydial EBs are not metabolically inert but maintain a limited metabolism during host-free survival (35, 36, 58, 61).

### Biphasic versus bipartite metabolism.

In summary, transcriptional dynamics during the *Protochlamydia* developmental cycle provide compelling evidence for a biphasic metabolism with stage-specific carbon and energy sources (Fig. 6). This is well supported by a number of earlier findings, suggesting that a biphasic metabolism is generally conserved among known chlamydiae (51, 58, 61).

In addition to these stage-specific differences in physiology, a recent study demonstrated cosubstrate usage by *C. trachomatis*, with host amino acids required for bacterial protein biosynthesis and glucose 6-phosphate as the carbon source for lipopolysaccharide biosynthesis (67). Termed bipartite metabolism, this efficient use of host-derived metabolites (i.e., the use of two different carbon sources) has also been observed in *Listeria monocytogenes* (70) and *Legionella pneumophila* (71) and might thus represent a more general adaptation of intracellular bacteria (67).

Yet, for *L. pneumophila*, there is also evidence for a growth phase-dependent metabolism. Intracellular growth of *L. pneumophila* within *A. castellanii* requires amino acids as energy source and—similarly to our model for *Protochlamydia*—as the carbon source and directly for protein biosynthesis (72, 73). Although a temporal separation of amino acid and carbohydrate usage has not yet been shown *in vivo* for *L. pneumophila*, a pronounced transcriptional switch between replicative and transmissive phase within *A. castellanii* has been reported (74). In addition, in *in vitro* experiments, *L. pneumophila* used serine as a major carbon source for the synthesis of other amino acids and for energy generation during all growth phases, while glucose served as the carbon source primarily in the postreplicative phase (71, 75). A growth or developmental stage-specific metabolism might thus not be restricted to the chlamydiae but is perhaps more widespread among intracellular bacteria.

### Transcriptional response of the *Acanthamoeba* host.

Close to 75% of the known *A. castellanii* genes (76) were detected to be expressed during infection with *Protochlamydia* (*n* = 12,044; Data Set S1). Although the different infection rates in our experiments (5 to 60%) entail capturing only a transcript mix of infected and uninfected amoebae, we still observed pronounced temporal patterns and characteristic expression profiles, with 3,582 *Acanthamoeba* genes found to be differentially expressed. Major transcriptional shifts occurred at 2 hpi when 1,722 genes were up- and subsequently downregulated and at 48 hpi when 1,747 genes were induced (Fig. S6).

10.1128/mSystems.00202-16.7 FIG S6  Gene expression dynamics of the *Acanthamoeba* host during infection with *Protochlamydia*. Ten different temporal subclasses of genes can be distinguished (indicated by black lines) within three main classes (colored bars). Major gene expression shifts occurred early during infection (yellow) and at the peak of *Protochlamydia* RB activity (red). Only significantly differentially expressed genes are shown. The number of genes per temporal class is indicated. RPKM, reads per kilobase per million; hpi, h postinfection. Download FIG S6, PDF file, 1.2 MB. Copyright © 2017 König et al. 2017 König et al. This content is distributed under the terms of the Creative Commons Attribution 4.0 International license.

Enrichment analysis of putative gene functions at the early stage of infection indicated a strong increase in cell signaling (serine threonine kinases), transport activity (ABC transporter of unknown specificity), translation (tRNA-related functions), and assembly of the mitochondrial respiratory chain complex III (Table S2). These effects on the *Acanthamoeba* host are consistent with the observed pronounced expression of type III secretion effectors by *Protochlamydia* at this stage, interfering with diverse host signaling pathways and possibly inducing a stress response.

10.1128/mSystems.00202-16.9 TABLE S2  Overrepresented functions among *A. castellanii* genes. Download TABLE S2, PDF file, 0.1 MB. Copyright © 2017 König et al. 2017 König et al. This content is distributed under the terms of the Creative Commons Attribution 4.0 International license.

When *Protochlamydia* is proliferating at the RB stage, the amoeba host transcriptome is characterized by increased expression of genes involved in breakdown of complex sugars (Table S2). This might account for the increased ATP demand of replicating *Protochlamydia* RBs. Notably downregulated at this stage are genes functioning in cell signaling (Ras proteins, serine threonine kinases), ubiquitination, translation (ribosome biogenesis), transcription (transcription factors), and replication (DNA replication initiation) (Table S2). Together, this suggests a general downregulation of central cellular processes due to replicating *Protochlamydia*, which is at the peak of its metabolic activity at this stage.

At later stages of the infection at which *Protochlamydia* EBs are formed, the amoeba transcriptome is still significantly altered and strikingly different compared to the onset of the infection. In particular, genes involved in cell signaling (histidine kinases, serine threonine kinases) are enriched at 96 hpi, whereas key cellular pathways are still less active, illustrating the fundamental impact of *Protochlamydia* on gene expression of its amoeba host (Fig. S6 and Table S2).

The response of human cells upon infection with *Chlamydiaceae* continues to be extensively studied (25, 77, 78), whereas studies on the impact of environmental chlamydiae on their host cells are strikingly lacking. Our transcriptomic data provide the first insights into this unexplored aspect of chlamydia-host interplay.

### Conclusions.

Owing to their obligate intracellular lifestyle and the lack of routine genetic methods (79), chlamydiae are—compared to many other bacterial pathogens—inherently difficult to study. This particularly applies to environmental chlamydiae, which were discovered only about 2 decades ago. The present study represents the most comprehensive analysis of the transcriptional landscape of chlamydiae during infection and development thus far. We obtained detailed insights into gene expression dynamics of *Protochlamydia* and present models for metabolism and the role and assembly of the type III secretion system at different developmental stages. Our findings revealed striking parallels to what is known about pathogenic chlamydiae and provide a substantially novel perspective on the interplay between chlamydiae and protist hosts. In addition, the vast majority of chlamydial organisms lie outside the well-known human and animal pathogens, and thus the *Protochlamydia* model system serves to represent this vast diversity. Environmental chlamydiae constitute invaluable model systems to understand fundamental chlamydial biology, common themes and differences among known chlamydiae, and to shed light on the evolution of the intracellular lifestyle and pathogenesis in a ubiquitous bacterial phylum.

## MATERIALS AND METHODS

### Cell culture.

*Acanthamoeba* *castellanii* Neff (ATCC 50373) with or without the symbiont *Protochlamydia amoebophila* UWE25 (ATCC PRA-7) were maintained at 20°C in PYG medium [20 g/liter proteose peptone, 100 mM glucose, 2 g/liter yeast extract, 1 g/liter sodium citrate dihydrate, 4 mM MgSO_4 _⋅_ _7H_2_O, 1.32 mM Na_2_HPO_4 _⋅ 2H_2_O, 2.5 mM KH_2_PO_4_, 0.05 mM Fe(NH_4_)_2_(SO_4_)_2_ ⋅ 6H_2_O; pH 6.5]. Cultures were regularly screened by fluorescence *in situ* hybridization and 4′,6′-diamidino-2-phenylindole (DAPI) staining (0.1 µg/ml) to exclude contamination.

### Infection experiments.

*Protochlamydia* EBs were freshly purified from amoeba cultures grown in 500-cm^2^ culture flasks (Nalge Nunc International, Rochester, NY, USA), in which EBs had been allowed to accumulate in the medium for 1 week. Purification of EBs was conducted by filtering culture supernatants through 5-µm and 1.2-µm syringe filters (Sartorius, Göttingen, Germany) to remove residual host cells. Bacteria were collected by centrifugation (15,550 × *g*, 15 min, 20°C), resuspended in precooled SPG buffer (75 g/liter sucrose, 0.52 g/liter KH_2_PO_4_, 1.53 g/liter NaHPO_4_ ⋅ 7H_2_O, 1.53 g/liter Na_2_HPO_4 _⋅ 2H_2_O, 0.75 g/liter glutamic acid; pH 7.2), homogenized using a 21-gauge injection needle (B. Braun, Melsungen, Germany), and stored overnight at 4°C in SPG buffer. For quantification of purified EBs, cell suspensions were filtered onto a polycarbonate membrane with a pore size of 0.2 µm (EMD Millipore, Billerica, MA, USA); cells were stained with DAPI and counted using an epifluorescence microscope (Axioplan 2 imaging; Carl Zeiss, Oberkochen, Germany).

Symbiont-free amoebae were harvested 3 days before infection and 6.4 × 10^7^ cells per 500-cm^2^ culture flask per time point and replicate were seeded and incubated at 20°C until infection. To optimize infection efficiency, particularly at early time points, we used a multiplicity of infection (MOI) of 150 for 2 h postinfection (hpi), an MOI of 100 for 48 hpi, and an MOI of 15 for 96 hpi. Precultivated amoebae were harvested, transferred to 50-ml Greiner tubes, and purified *Protochlamydia* EBs were added, followed by repeated centrifugation (centrifuged at 130 × *g* twice for 5 min each time and then once for 10 min at 20°C) with vortexing between the centrifugation steps. Infected amoebae were then transferred back to the culture flasks and incubated in PYG medium at 20°C for 2 h before the infection was synchronized by gently washing the attached amoebae three times with Page’s amoebic saline (PAS) (0.12 g/liter NaCl, 0.004 g/liter MgSO_4_ ⋅ 7H_2_O, 0.004 g/liter CaCl_2_ ⋅ 2H_2_O, 0.142 g/liter Na_2_HPO_4_, 0.136 g/liter KH_2_PO_4_). PYG medium was added to the cultures, the culture was sampled at the 2 hpi time point, and the remaining culture flasks were incubated at 20°C for 48 and 96 h. Extracellular *Protochlamydia* EBs were purified as described above. All infection experiments were performed in biological triplicates. One culture of symbiont-free amoebae was harvested when the culture was sampled at 2 hpi.

### Fluorescence *in situ* hybridization and transmission electron microscopy.

Fluorescence *in situ* hybridization using a combination of two Cy3-labeled probes (Chls-0523, E25-454; Thermo Fisher Scientific, Waltham, MA, USA) was performed as described elsewhere (35, 80, 81), and cells were stained with DAPI for 5 min. Images were recorded with a charge-coupled-device (CCD) camera (AxioCam HRc; Carl Zeiss) connected to an epifluorescence microscope and were processed using the AxioVision 4.6.3 software package (Carl Zeiss).

For transmission electron microscopy, the culture medium was replaced with fixative solution (2.5% glutaraldehyde in 3 mM cacodylate containing 0.1 M sucrose; pH 6.5). Amoebae were fixed for 1 h at room temperature, then collected, washed three times (0.1 M cacodylate containing 0.1 M sucrose [pH 7.2]), and mixed with one drop of 1% Biozym plaque agarose (Biozym, Hessisch Oldendorf, Germany) in washing buffer equilibrated at 35°C. Secondary fixation was conducted in 1% buffered osmium tetroxide for 1 h on ice, followed by dehydration in ethanol and infiltration with low-viscosity resin (Agar Scientific, Essex, United Kingdom). Ultrathin sections (70 nm) were cut using a Leica EM UC7 ultramicrotome (Leica, Wetzlar, Germany) and stained with 0.5% uranyl acetate and 3% lead citrate, and imaging was done with a Zeiss EM 902 transmission electron microscope (Carl Zeiss).

### Infectious progeny production assay.

To monitor the production of infectious *Protochlamydia* EBs during the developmental cycle, amoebae were infected with *Protochlamydia* using an MOI of 10. The infection was synchronized by centrifugation (130 × *g*, 15 min, 20°C) and subsequent medium exchange. Cells were harvested at different time points postinfection, and extracellular bacteria were separated from amoebae by low-speed centrifugation (300 × *g*, 10 min, 4°C). The supernatant containing the extracellular bacteria was centrifuged (20,800 × *g*, 30 min, 4°C), the pellet was resuspended in precooled SPG medium, and stored at −80°C until further use. The pellet containing the infected amoebae was resuspended in precooled SPG medium and subjected to two freeze/thaw (−20°C/room temperature) steps, followed by vortexing with glass beads (diameter of 0.75 to 1 mm; Carl Roth, Karlsruhe, Germany) for 3 min to break up the amoebae. Amoeba cell debris and glass beads were removed by centrifugation (300 × *g*, 10 min, 4°C), and the supernatants containing intracellular bacteria were stored at −80°C. The total numbers of bacteria in intra- and extracellular suspensions were determined by counting DAPI signals as described above. The percentage of infectious *Protochlamydia* EBs was determined by infecting fresh amoebae and counting bacteria (inclusions) per amoeba at 12 hpi. The infection was carried out as described above. Bacteria were detected by indirect immunofluorescence and DAPI staining after methanol fixation as described previously (82). Mean relative infectivity of each fraction (intra/extracellular) per time point was expressed as the number of bacteria or amoeba/total number of bacteria (± standard error of the mean, three biological replicates).

### RNA extraction and sequencing.

Preliminary tests showed that RNA extraction and sequencing of intact *Protochlamydia*-infected amoebae yielded an insufficient number of bacterial transcripts. Thus, to increase the coverage of the *Protochlamydia* transcriptome, a protocol for enrichment of bacteria prior to RNA extraction was developed. To minimize possible changes of the transcriptomes during enrichment, each sample was processed in less than 7 min, as the half-life of total mRNA from *Escherichia coli* was demonstrated to be in this range (83). Infected amoebae were harvested and collected (7,600 × *g*, 2 min, 20°C), and the pellets were resuspended in a sucrose buffer (35 mM Tris-HCl, 250 mM sucrose, 25 mM KCl, 10 mM MgCl_2_) supplemented with 50 µg/ml rifampin in order to inhibit active transcription during the enrichment procedure (84, 85). Amoebae were then disrupted by vortexing in the presence of glass beads (diameter of 0.75 to 1 mm; Carl Roth) for 1 min. The suspensions were subsequently filtered through a 5-µm filter, the flowthrough fractions containing the bacteria were collected by centrifugation (10,600 × *g*, 2 min, room temperature), and the pellets were immediately resuspended in TRIzol reagent (Thermo Fisher Scientific). Extracellular *Protochlamydia* EBs were pelleted (20,800 × *g*, 2 min, room temperature), and the pellets were resuspended in sucrose buffer and subsequently treated like the enriched bacteria.

Cells were mechanically disrupted by beat beating for 30 s at 4.5 m/s using lysing matrix A tubes and a FastPrep-24 instrument (MP Biomedicals, Santa Ana, CA, USA). Subsequent RNA extraction was performed according to the TRIzol guidelines. Residual DNA was digested using the Turbo DNA-free kit (Thermo Fisher Scientific) according to the manufacturer’s instructions. DNase-treated RNA was precipitated with ethanol and sodium acetate and dissolved in nuclease-free water (Thermo Fisher Scientific), and DNA contamination was controlled for via PCR targeting a short region of the bacterial 16S rRNA gene (SigF2/R2 primers; 11) using 35 PCR cycles. rRNA was removed using the Ribo-Zero magnetic kit for Gram-positive bacteria as recommended by the manufacturer (Illumina, San Diego, CA, USA). To enrich for mRNA from symbiont-free amoebae, the RNA was additionally treated with Dynabeads mRNA purification kit (Thermo Fisher Scientific). After another round of precipitation with ethanol, rRNA depletion and RNA quality were examined using the Experion automated electrophoresis system (Bio-Rad Laboratories, Hercules, CA, USA). RNA fragmentation was performed at 70°C for 5 min using the RNA fragmentation reagents from Thermo Fisher Scientific and was followed by another ethanol precipitation. For strand-specific cDNA library preparation, the NEBNext Ultra directional RNA library prep kit for Illumina in combination with the NEBNext multiplex oligonucleotides (New England Biolabs, Ipswich, MA, USA) was used starting at first-strand cDNA synthesis. Purification and size selection steps were done as recommended using Agencourt AMPure beads (Beckman Coulter, Brea, CA, USA). All libraries were sequenced using an Illumina HiSeq2000 system at the Vienna Biocenter Core Facilities (VBCF) Next-Generation Sequencing (NGS) Unit (http://www.vbcf.ac.at) with 50-bp read length.

### Sequence read processing.

Sequencing reads were trimmed and cleaned before mapping (see Text S1 in the supplemental material). To map bacterial reads to the *Protochlamydia* genome (18), the Burrows-Wheeler Aligner (BWA) (86) was used; amoeba reads were mapped to the *A. castellanii* Neff genome (76), the rRNA genes (87–89), and the mitochondrial genome (90) using TopHat (91), both with default settings. Only unambiguously mapped reads were kept using SAMtools (92). Strand-specific reads per predicted gene were counted via HTSeq (93). Reads that could not be assigned to any gene but mapped to the genome were considered transcripts of intergenic regions (IGRs) and antisense transcripts.

### Gene expression analyses.

Differentially expressed genes were determined between two consecutive time points (2 hpi to 48 hpi, 48 hpi to 96 hpi, 96 hpi to extracellular, extracellular to 2 hpi for *Protochlamydia*; uninfected to 2 hpi, 2 hpi to 48 hpi, 48 hpi to 96 hpi for *A. castellanii*) using the R software environment and the Bioconductor package edgeR (94–96). Genes were considered differentially expressed if their expression changed twofold with a false-discovery rate (FDR) smaller or equal 0.05, except for detecting gene expression changes between uninfected amoebae and infected amoebae 2 hpi, when a fivefold change threshold was used because only one sample of uninfected amoebae was sequenced.

Gene expression data were further analyzed using custom R scripts, integrated R tools, and R packages (94). To determine temporal expression patterns, sets of genes with similar expression profiles were identified by hierarchical clustering of gene expression values (log_2 _reads per kilobase per million [RPKM]) based on Pearson correlation distances. Obtained clusters were validated using the R package clValid (97). Mean centered expression values were used for visualization as heatmaps using the R package gplots (98).

To extend and improve the available *Protochlamydia* genome annotation by Horn et al. (18) for each gene, we collected Pfam domains (99), Kyoto Encyclopedia of Genes and Genomes (KEGG) pathway and gene ontology (GO) term level 5 assignments using DAVID (100), COG (clusters of orthologous groups of proteins) cluster and class assignments using MaGe (101), and type III secretion effector predictions using Effective (102). Blast2GO (103) was used to assign GO terms to predicted proteins of *A. castellanii*. The Bioconductor software package GOseq (104) and the Blast2GO enrichment analysis tool were used to test for statistical enrichment of functional categories (FDR < 0.05) among differentially expressed genes per time point or temporal class. To test whether predicted type III secreted proteins were significantly enriched in any given gene set, two-tailed Fisher’s exact tests were conducted, and *P* values below 0.05 were considered statistically significant.

### Availability of data.

Sequences were deposited at the Gene Expression Omnibus (GEO) database and are accessible through accession number GSE93891.
