# Supplementary material for: Biphasic Metabolism and Host Interaction of a Chlamydial Symbiont
Source: mSystems. 2017 May 30;2(3):e00202-16. doi: 10.1128/mSystems.00202-16 (PMC5451489; doi:10.1128/mSystems.00202-16)
Supplement: TEXT S1 [file sys003172105s10.pdf]

Text S1. Supplemental Methods, Results and Discussion

The *Protochlamydia amoebophila* developmental cycle. To identify key time points in the developmental cycle of *P. amoebophila* in *Acanthamoeba castellanii*, we monitored the course of infection using FISH, DAPI staining, and TEM. In addition, we quantified the production of infectious EBs.

Early after infection (2 hpi) most intracellular bacteria were only detected with DAPI but not with FISH probes, suggesting that the majority of *P. amoebophila* still resembled EBs, which are not easily identified by FISH due to their rigid cell wall but are readily visible with the DNA dye DAPI (Fig. 1A, 1). Some bacterial cells were stained by both FISH and DAPI, indicating that they had already started to convert to RBs (which are more accessible for the oligonucleotide probes used in FISH, 1). These observations are consistent with the cell morphologies detected by TEM (Fig. 1C). *P. amoebophila* at this early stage were no longer infectious in an infection assay (Fig. 1B), indicating that the majority of cells already initiated differentiation to RBs. *P. amoebophila* in contrast to other chlamydiae generally reside in single-cell inclusions (2, 3), but at 2 hpi inclusions containing multiple bacteria were found (Fig. 1C). Accumulation of membrane structures was observed in some inclusions, which might represent remnants of bacterial cells (Fig. 1C).

At 24 and 48 hpi all bacteria were detected with both FISH and DAPI, indicating that EBs had converted to RBs. Cell division begins around 24 hpi as demonstrated by brighter FISH signals and the detection of constricting cells (inset in Fig. 1A) whereas the number of intracellular bacteria had not yet increased. Consistent with this, dividing cells were first detected at 24 hpi by TEM (Fig. 1C). Small outer membrane vesicles in the inclusion lumen

indicating transport activity (4–7) were also first seen at this stage (Fig. 1C). The enlarged FISH signals, dividing cells in TEM and an increased number of bacteria per amoeba at 48 hpi suggest that at this time point all RBs were dividing (Figs. 1A, C). Conversion from RBs back to EBs started between 48 and 72 hpi with the remaining cells still dividing: bacteria only detected with DAPI were first visible at 72 hpi (Fig. 1A), and first bacterial cells with more electron dense nucleoids appeared at this time point (Fig. 1C), while cell division could still be seen (Fig. 1C). The onset of secondary differentiation is also supported by a slightly increased proportion of infectious intracellular particles at 72 hpi (Fig. 1B).

The number of EBs within amoebae further increases at 96 hpi, as shown by a greater percentage of DAPI-only signals (Fig. 1A) and a peak in the proportion of infectious intracellular particles (Fig. 1B). In addition, extracellular bacteria could be seen by TEM (Fig. 1C) pointing at a first release of infectious EBs (Fig. 1B) and therefore completion of the cycle at 96 hpi. As RBs were still present, this late time point represents an asynchronous stage in the developmental cycle in which EBs predominate (8). The number of released bacteria further increased until 120 hpi, and a higher proportion of extracellular EBs was infectious at this time point (Fig. 1B). Lysis of *Acanthamoeba* host cells was not observed indicating that *P. amoebophila* primarily exits the host cells in a non-lytic manner (9).

In summary, *P. amoebophila* in its *Acanthamoeba* host exhibits the characteristic chlamydial developmental cycle, including a rapid loss of infectivity after uptake (10, 11), a highly synchronous stage during which RBs undergo several rounds of cell division, secondary differentiation into EBs and release at the end. Completion of the cycle takes with 96h slightly longer than what has been reported for chlamydiae infecting humans and animals, e.g. *C.*

trachomatis (in L929 cells, 2-3 days; 12), *C. pneumoniae* (in HeLa cells, 3 days; 13) and *Waddlia chondrophila* (in macrophages, 2-3 days; 14). It is, however, shorter than those of *Parachlamydia acanthamoeba* (in *Acanthamoeba polyphaga*, 6 days; 15), *Rhabdochlamydia porcellionis* (in insect cells, 7 days; 16) and *Simkania negevensis* (in Vero cells, 12-15 days; 17). A characteristic feature of *P. amoebophila* infection is the presence of single-cell vacuoles (Fig. 1C), which imply that inclusions divide along with dividing RBs – a curious process that has not been described for other chlamydiae, but has been observed for other intracellular bacteria (18–20). Furthermore, *P. amoebophila* is less lytic than other chlamydiae; they co-exist with their amoeba hosts and can be maintained in long-term co-culture.

Sequencing statistics. Between 73,000 and 74 million high quality sequence reads could be mapped to the *P. amoebophila* genome (Table S1). The pronounced differences between time points are mainly caused by differences in bacterial load, reflecting the technical challenge to obtain a high transcriptome coverage of intracellular bacteria early in infection (21). Still, the coverage achieved here is comparable to a recent *C. trachomatis* study (22), and sufficient as the number of expressed genes detected was almost identical for all time points (1,476-1,518 genes corresponding to 71-73% of all predicted genes; Fig. S1). The expression values were highly congruent between biological replicates as demonstrated by low Euclidean distances between normalized gene expression values (RPKMs) and grouping in a hierarchical cluster analysis (Fig. S1). This was less pronounced only for the early time point due to the lower sequencing depth and a consequently less homogeneous distribution of reads across the three replicates. The grouping by time point not only highlights reproducibility but also indicates

distinct gene expression profiles, were 48 hpi, 96 hpi and the extracellular stage were more similar to each other than the 2 hpi time point, suggesting major transcriptional changes during early development (Fig. S1).

Gene expression dynamics. Analyzing the number of significantly up- or downregulated genes per time point provided a more detailed picture of the expression dynamics during the developmental cycle (Fig. S3). The largest number of genes were significantly regulated (n = 493) – either induced (n = 250) or shut down (n = 243) – upon host entry at 2 hpi (Fig. S3). Thus, the transition to the intracellular environment requires a high degree of adjustment. The largest shutdown of genes occurred between early and mid-cycle, with 279 genes (or more than one third of all DE genes) being downregulated at 48 hpi (Fig. S3). This major gene expression switch was underscored by 153 genes that were significantly upregulated early and subsequently downregulated at mid-cycle (Fig. S3, orange gene set). Genes encoding for putatively secreted proteins were overrepresented in this gene set, suggesting that interaction with the host is a hallmark of early development. Once the intracellular niche is established and replication has started, host manipulation is strongly reduced. The smallest change in gene expression occurred between 96 hpi and the extracellular stage, with 74 genes significantly upregulated and only 16 genes downregulated after host cell exit (Fig. S3), demonstrating that most of the late transcripts were also detected in extracellular EBs.

In conclusion, major regulatory shifts occurred during entry, establishment of the intracellular niche and EB to RB differentiation. This is notably different for the *Chlamydiaceae* where the largest group of DE genes was observed at mid-cycle (23). As the absolute number of

mid-cycle genes is comparable between *P. amoebophila*, *C. trachomatis* (12) and *C. pneumoniae* (13), *Protochlamydia* does not require fewer genes at the RB stage, but instead employs a larger number of transcriptionally active genes early during infection. Compared to these initial events, secondary differentiation of *Protochlamydia* from RB to EB, and host cell exit is accompanied by fewer regulatory events.

Analyzing the distribution of conserved and species-specific genes across the observed temporal gene expression classes. To investigate the evolutionary conservation of differentially regulated genes, groups of orthologous chlamydial proteins determined previously using OrthoMCL (24) were first used to classify *Protochlamydia* genes into strain-specific (*P. amoebophila* UWE25), species-specific (*P. amoebophila* strains UWE25 and EI2), family-specific (*Protochlamydia, Parachlamydia, Neochlamydia* species), or environmental chlamydiae-specific genes (*Protochlamydia*, *Parachlamydia*, *Neochlamydia* species, *Waddlia chondrophila*, *Simkania negevensis*), or genes found in all chlamydiae (for a detailed list see 24). Next, we analyzed the distribution of these conserved and species-specific genes across the observed temporal gene expression classes (Fig. S5).

Strikingly, the early gene set showed a high proportion of *Protochlamydia*-specific genes. This was even more pronounced for a subset of these genes, which were highly expressed not only at 2 hpi but also at the EB stage (termed early I in Fig. 2) and which comprise nearly 50% species-specific genes. As genes shared by chlamydiae that are able to thrive in amoebae do not substantially contribute to the early gene set, invasion of *Acanthamoeba* seems to be particularly guided by species-specific genes. It has been suggested before that amoeba-

associated chlamydiae use different invasion strategies than *Chlamydiaceae* (24–26), but it is surprising that genes expressed by *Protochlamydia* during host entry are not conserved among amoeba-associated chlamydiae. This indicates that different strategies for the infection of amoeba hosts exist among environmental chlamydiae.

Ninety-seven percent of the genes conserved among all chlamydiae were expressed during the developmental cycle (Fig. S5). More than half of those were transcribed constitutively, which is consistent with them encoding housekeeping functions (25). Also, all well conserved chlamydial transcription factors identified in previous studies (27, 28) were expressed, confirming their role as key genes controlling cellular transcription levels during chlamydial development. Chlamydial core genes dominated the mid-cycle gene set, confirming their role in RB metabolism and replication (Fig. S5, 25). Taken together, RB activity and proliferation are well conserved between amoeba-associated chlamydia and the *Chlamydiaceae*. Our data thus lend experimental support to genome-based hypotheses suggesting distinct roles for species-specific and chlamydial core genes (24–27, 29).

Sequence read trimming and cleaning workflow. In order to improve mapping and reduce the amount of data, sequencing reads were subjected to a 6-step cleaning workflow based on sequencing read statistics obtained using PRINSEQ-lite (30) and FastQC (31). Briefly, (i) the first 13 bases were removed due to a GC bias caused by using random hexamers during library preparation (PRINSEQ-lite), (ii) the reads were trimmed using mothur (32) when the base calling quality decreased below a phred score of 20 within a window of 10 bases, (iii) reads containing more than two ambiguous bases (Ns) were removed (PRINSEQ-lite), (iv) poly-A/T tails were

removed (PRINSEQ-lite), (v) any remaining adapter sequences were clipped using tools from Biopieces (http://maasha.github.io/biopieces/), and finally (vi) sequence reads shorter than 25 bases were removed (PRINSEQ-lite).

Supplemental References

1. **Poppert S**, **Essig A**, **Marre R**, **Wagner M**, **Horn M**. 2002. Detection and differentiation of chlamydiae by fluorescence in situ hybridization. Appl Environ Microbiol **68**:4081–4089.

2. **Collingro A**, **Toenshoff E**, **Taylor M**, **Fritsche T**, **Wagner M**, **Horn M**. 2005. “Candidatus Protochlamydia amoebophila”, an endosymbiont of Acanthamoeba spp. Int J Syst Evol Microbiol **55**:1863–1866.

3. **Pilhofer M**, **Aistleitner K**, **Ladinsky MS**, **König L**, **Horn M**, **Jensen GJ**. 2014. Architecture and host interface of environmental chlamydiae revealed by electron cryotomography. Environ Microbiol **16**:417–429.

4. **Stirling P**, **Richmond SJ**. 1980. Production of outer membrane blebs during chlamydial replication. FEMS Microbiol Lett **9**:103–105.

5. **Richmond SJ**, **Stirling P**. 1981. Localization of chlamydial group Antigen in McCoy cell monolayers infected with Chlamydia trachomatis or Chlamydia psittaci. Infect Immun **34**:561–570.

6. **Giles DK**, **Whittimore JD**, **LaRue RW**, **Raulston JE**, **Wyrick PB**. 2006. Ultrastructural analysis of chlamydial antigen-containing vesicles everting from the Chlamydia trachomatis inclusion. Microbes Infect **8**:1579–1591.

7. **Frohlich KM**, **Hua Z**, **Quayle AJ**, **Wang J**, **Lewis ME**, **Chou C**, **Luo M**, **Buckner LR**, **Shen L**. 2014. Membrane vesicle production by Chlamydia trachomatis as an adaptive response. Front Cell Infect Microbiol **4**:73.

8. **Haider S**, **Wagner M**, **Schmid MC**, **Sixt BS**, **Christian JG**, **Häcker G**, **Pichler P**, **Mechtler K**, **Müller A**, **Baranyi C**, **Toenshoff ER**, **Montanaro J**, **Horn M**. 2010. Raman microspectroscopy reveals long-term extracellular activity of Chlamydiae. Mol Microbiol **77**:687–700.

9. **Hybiske K**, **Stephens RS**. 2007. Mechanisms of host cell exit by the intracellular bacterium Chlamydia. Proc Natl Acad Sci U S A **104**:11430–11435.

10. **Shaw EI**, **Dooley CA**, **Fischer ER**, **Scidmore MA**, **Fields KA**, **Hackstadt T**. 2000. Three temporal classes of gene expression during the Chlamydia trachomatis developmental cycle. Mol Microbiol **37**:913–925.

11. **Moulder JW**. 1991. Interaction of chlamydiae and host cells in vitro. Microbiol Rev **55**:143–190.

12. **Nicholson TL**, **Olinger L**, **Chong K**, **Schoolnik G**, **Stephens RS**. 2003. Global stage-specific gene regulation during the developmental cycle of Chlamydia trachomatis. J Bacteriol **185**:3179–3189.

13. **Mäurer AP**, **Mehlitz A**, **Mollenkopf HJ**, **Meyer TF**. 2007. Gene expression profiles of Chlamydophila pneumoniae during the developmental cycle and iron depletion-mediated persistence. PLoS Pathog **3**:e83.

14. **Goy G**, **Croxatto A**, **Greub G**. 2008. Waddlia chondrophila enters and multiplies within

human macrophages. Microbes Infect **10**:556–562.

15. **Greub G**, **Raoult D**. 2002. Crescent bodies of Parachlamydia acanthamoeba and its life cycle within Acanthamoeba polyphaga: an electron micrograph study. Appl. Environ. Microbiol. **68**:3076–3084.

16. **Sixt BS**, **Kostanjšek R**, **Mustedanagic A**, **Toenshoff ER**, **Horn M**. 2013. Developmental cycle and host interaction of Rhabdochlamydia porcellionis, an intracellular parasite of terrestrial isopods. Environ Microbiol **15**:2980–2993.

17. **Kahane S**, **Kimmel N**, **Friedman MG**. 2002. The growth cycle of Simkania negevensis. Microbiology **148**:735–742.

18. **Baumann P**, **Baumann L**, **Lai CY**, **Rouhbakhsh D**, **Moran NA**, **Clark MA**. 1995. Genetics, physiology, and evolutionary relationships of the genus Buchnera: intracellular symbionts of aphids. Annu Rev Microbiol **49**:55–94.

19. **Douglas AE**. 2003. Buchnera bacteria and other symbionts of aphids, p. 368. *In* Bourtzis, K, Miller, TA (eds.), Insect symbiosis. CRC Press.

20. **Di Russo Case E**, **Smith JA**, **Ficht TA**, **Samuel JE**, **de Figueiredo P**. 2016. Space: a final frontier for vacuolar pathogens. Traffic **17**:461–474.

21. **Westermann AJ**, **Gorski SA**, **Vogel J**. 2012. Dual RNA-seq of pathogen and host. Nat Rev Microbiol **10**:618–630.

22. **Humphrys MS**, **Creasy T**, **Sun Y**, **Shetty AC**, **Chibucos MC**, **Drabek EF**, **Fraser CM**, **Farooq U**, **Sengamalay N**, **Ott S**, **Shou H**, **Bavoil PM**, **Mahurkar A**, **Myers GSA**. 2013. Simultaneous Transcriptional Profiling of Bacteria and Their Host Cells. PLoS One

8:e80597.

23. **Tan M**. 2012. Temporal gene regulation during the chlamydial developmental cycle, p. . *In* Tan, M, Bavoil, P (eds.), Intracellular Pathogens I: Chlamydiales, 10thed. John Wiley & Sons.

24. **Domman D**, **Collingro A**, **Lagkouvardos I**, **Gehre L**, **Weinmaier T**, **Rattei T**, **Subtil A**, **Horn M**. 2014. Massive Expansion of Ubiquitination-Related Gene Families within the Chlamydiae. Mol Biol Evol **31**:2890–2904.

25. **Collingro A**, **Tischler P**, **Weinmaier T**, **Penz T**, **Heinz E**, **Brunham RC**, **Read TD**, **Bavoil PM**, **Sachse K**, **Kahane S**, **Friedman MG**, **Rattei T**, **Myers GSA**, **Horn M**. 2011. Unity in variety--the pan-genome of the Chlamydiae. Mol Biol Evol **28**:3253–3270.

26. **Aistleitner K**, **Anrather D**, **Schott T**, **Klose J**, **Bright M**, **Ammerer G**, **Horn M**. 2015. Conserved features and major differences in the outer membrane protein composition of chlamydiae. Environ Microbiol **17**:1397–1413.

27. **Domman D**, **Horn M**. 2015. Following the Footsteps of Chlamydial Gene Regulation. Mol Biol Evol **32**:3035–3046.

28. **de Barsy M**, **Frandi A**, **Panis G**, **Théraulaz L**, **Pillonel T**, **Greub G**, **Viollier PH**. 2016. Regulatory (pan-)genome of an obligate intracellular pathogen in the PVC superphylum. ISME J **10**:2129–2144.

29. **Heinz E**, **Rockey DD**, **Montanaro J**, **Aistleitner K**, **Wagner M**, **Horn M**. 2010. Inclusion membrane proteins of Protochlamydia amoebophila UWE25 reveal a conserved mechanism for host cell interaction among the Chlamydiae. J Bacteriol **192**:5093–5102.

30. **Schmieder R**, **Edwards R**. 2011. Quality control and preprocessing of metagenomic datasets. Bioinformatics **27**:863–864.

31. **Andrews S**. 2010. FastQC: a quality control tool for high throughput sequence data.

32. **Schloss PD**, **Westcott SL**, **Ryabin T**, **Hall JR**, **Hartmann M**, **Hollister EB**, **Lesniewski RA**, **Oakley BB**, **Parks DH**, **Robinson CJ**, **Sahl JW**, **Stres B**, **Thallinger GG**, **Van Horn DJ**, **Weber CF**. 2009. Introducing mothur: open-source, platform-independent, community-supported software for describing and comparing microbial communities. Appl Environ Microbiol **75**:7537–7541.
