# Supplementary material for: Biphasic Metabolism and Host Interaction of a Chlamydial Symbiont
Source: mSystems. 2017 May 30;2(3):e00202-16. doi: 10.1128/mSystems.00202-16 (PMC5451489; doi:10.1128/mSystems.00202-16)
Supplement: FIG S1 [file sys003172105sf1.pdf]

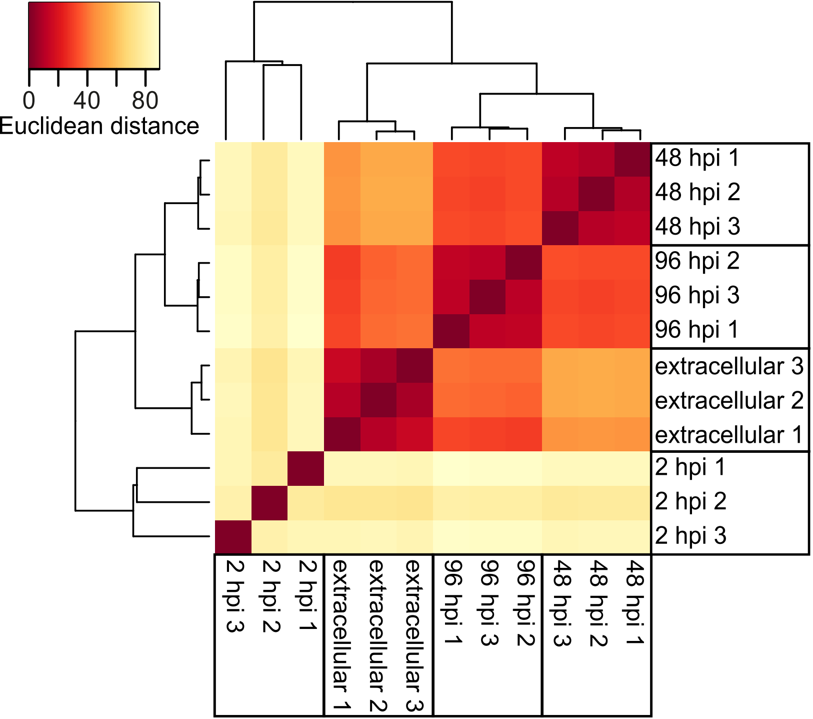

2,066 predicted genes (excl. 9 rRNA genes)
1,518 genes expressed (in total)

Expressed per time point	Not expressed in only one replicate
	0
1,517	0
	1
	0
1,518	1
	2
	0
1,518	0
	0
	46
1,476	39
	62
