# Supplementary figures and images for: Biphasic Metabolism and Host Interaction of a Chlamydial Symbiont
Source: mSystems. 2017 May 30;2(3):e00202-16. doi: 10.1128/mSystems.00202-16 (PMC5451489; doi:10.1128/mSystems.00202-16)

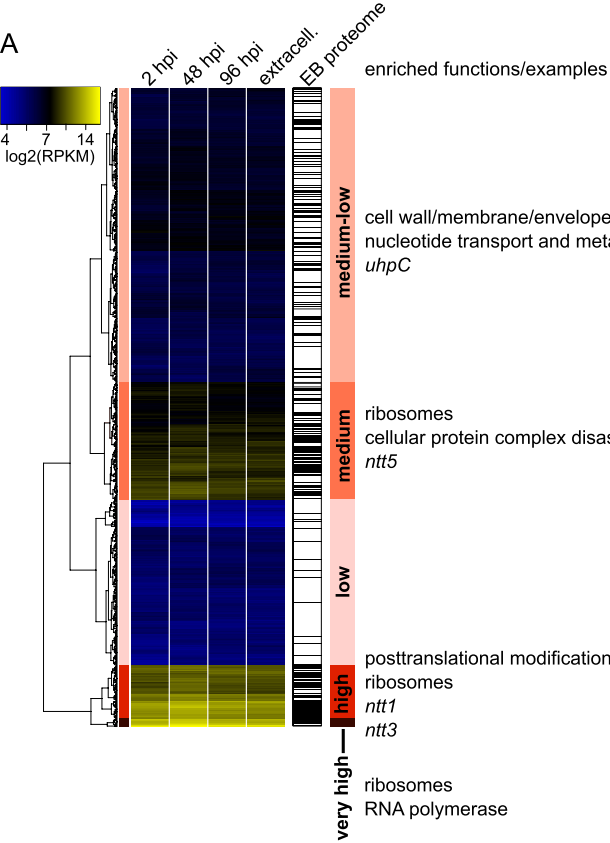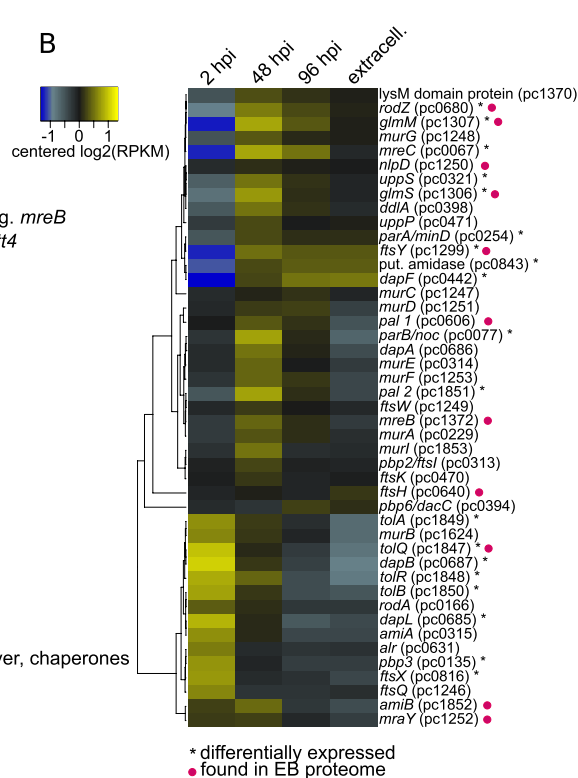

Supplement: FIG S2 [file sys003172105sf2.pdf]

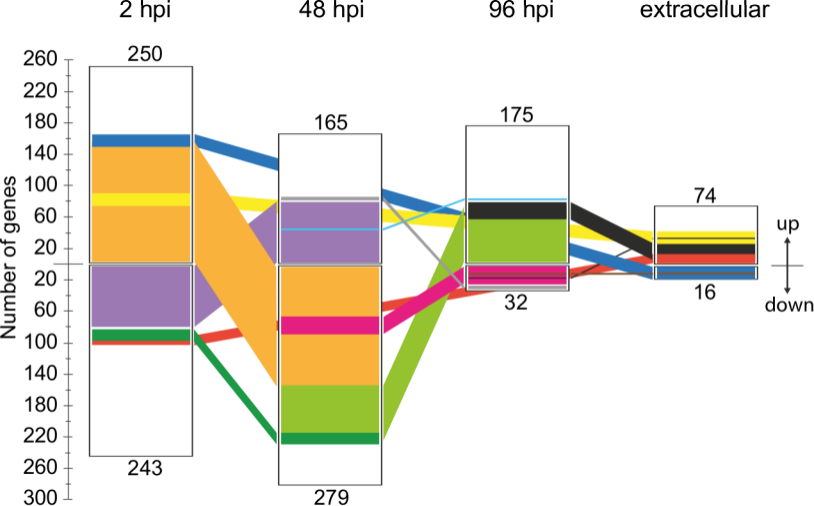

Supplement: FIG S3 [file sys003172105sf3.pdf]

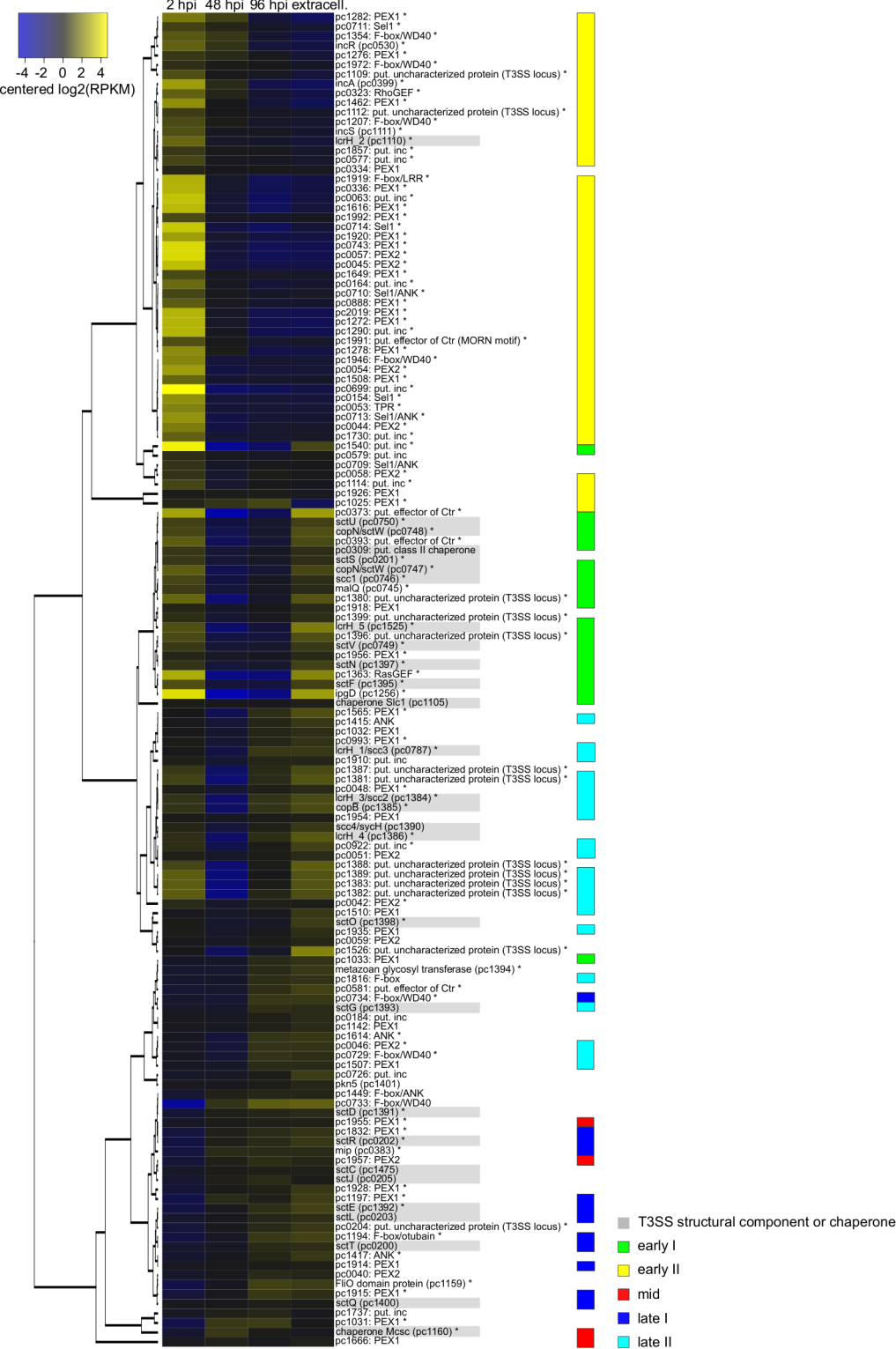

Supplement: FIG S4 [file sys003172105sf4.pdf]

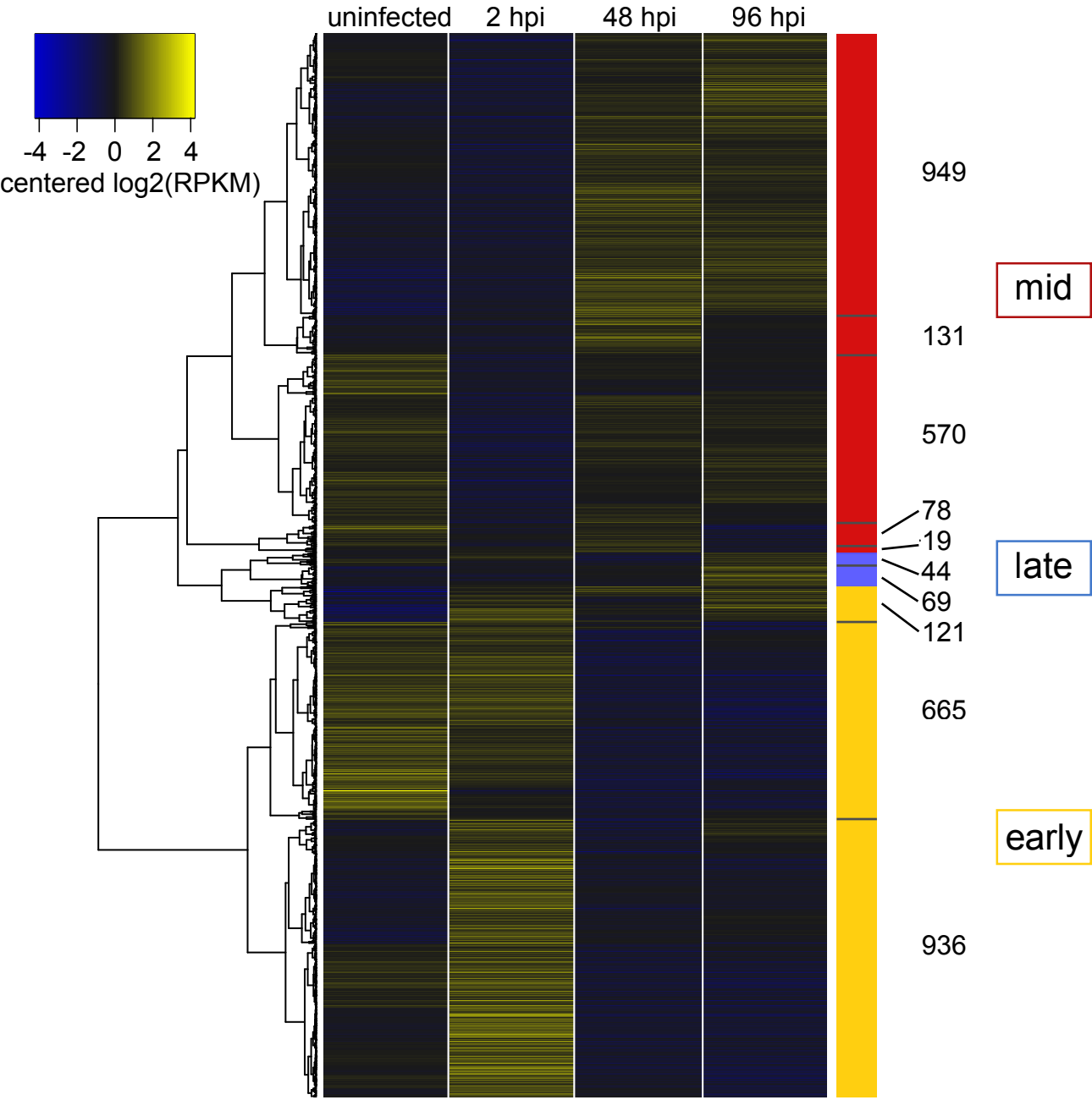

Supplement: FIG S6 [file sys003172105sf6.pdf]
