# Supplementary material for: Biphasic Metabolism and Host Interaction of a Chlamydial Symbiont
Source: mSystems. 2017 May 30;2(3):e00202-16. doi: 10.1128/mSystems.00202-16 (PMC5451489; doi:10.1128/mSystems.00202-16)
Supplement: TABLE S1 [file sys003172105st8.pdf]

Table S1. Sequencing and read mapping statistics

	2 hpi*	48 hpi*	96 hpi*	extracell.*	symbiont -free amoebae
Total reads[#]	217 ± 5.9	222 ± 14	175 ± 18	218 ± 19	57
Reads after cleaning[#†]	215 ± 5.2	219 ± 12	174 ± 18	216 ± 19	53
P. amoebophila					
Mapped to genome[#‡]<br>% of cleaned	0.073 ± 0.025<br>0.03 ± 0.01	1.7 ± 0.58<br>0.78 ± 0.2	0.77 ± 0.15<br>0.44 ± 0.05	74 ± 9.5<br>34.3 ± 2.0	n.a.
Mapped to predicted genes[#]<br>% of mapped	0.044 ± 0.015<br>61.0 ± 1.0	1.4 ± 0.47<br>80.1 ± 0.1	0.59 ± 0.1<br>76.2 ± 1.0	42 ± 7.3<br>56.1 ± 2.8	n.a.
Mapped to IGRs and antisense[#]<br>% of mapped	0.028 ± 0.01<br>38.2 ± 1.2	0.32 ± 0.11<br>18.6 ± 0.1	0.18 ± 0.042<br>22.7 ± 1.0	32 ± 2.6<br>43.2 ± 2.9	n.a.
Mapped to rRNA genes<br>% of mapped	30.7 ± 17.0<br>0.05 ± 0.04	1886.3 ± 2224.3<br>0.09 ± 0.09	45.0 ± 33.2<br>0.006 ± 0.005	0.21 ± 0.13<br>0.3 ± 0.2	n.a.
A. castellanii					
Mapped to chromosome[#§]<br>% of cleaned	98 ± 11<br>45.4 ± 4.6	74 ± 2.8<br>33.8 ± 2.5	64 ± 5.1<br>36.6 ± 3.4	n.a.	23<br>43.1
Mapped to predicted chr. genes[#]<br>% of mapped	7.8 ± 1.3<br>8.0 ± 1.6	9 ± 0.73<br>12.2 ± 1.0	6 ± 1.1<br>9.5 ± 1.8	n.a.	14<br>59.7
Mapped to chr. IGRs and antisense[#]<br>% of mapped	29.4 ± 4.1<br>30.3 ± 4.7	17.2 ± 2.5<br>23.3 ± 3.1	14.2 ± 2.2<br>22.3 ± 1.9	n.a.	2.0<br>8.7
Mapped to mitoch. genome[#]<br>% of cleaned	11 ± 2.1<br>5.2 ± 1.0	4.5 ± 1.8<br>2.0 ± 0.7	3.7 ± 0.6<br>2.1 ± 0.1	n.a.	0.12<br>0.2
Mapped to rRNA genes (chromosome, mitoch. genome)[#]<br>% of mapped total	61 ± 12<br>55.7 ± 7.1	48 ± 3.5<br>61 ± 5.0	43 ± 3.5<br>64.5 ± 3.5	n.a.	7.3<br>31.5

* All numbers are means and standard deviations of three biological replicates.
[#] Numbers multiplied by 10^6.
[†] The read lengths range from 25 to 37 bp (average 36.7 ± 1.1 bp).
[‡] Ambiguously mapped reads removed.
[§] Including chromosomal rRNA.
Abbreviations: hpi, hours post infection; extracell., extracellular; IGRs, intergenic regions; chr., chromosomal; mitoch., mitochondrial; n.a., not applicable.
