# Supplementary material for: Biphasic Metabolism and Host Interaction of a Chlamydial Symbiont
Source: mSystems. 2017 May 30;2(3):e00202-16. doi: 10.1128/mSystems.00202-16 (PMC5451489; doi:10.1128/mSystems.00202-16)
Supplement: FIG S5 [file sys003172105sf5.pdf]

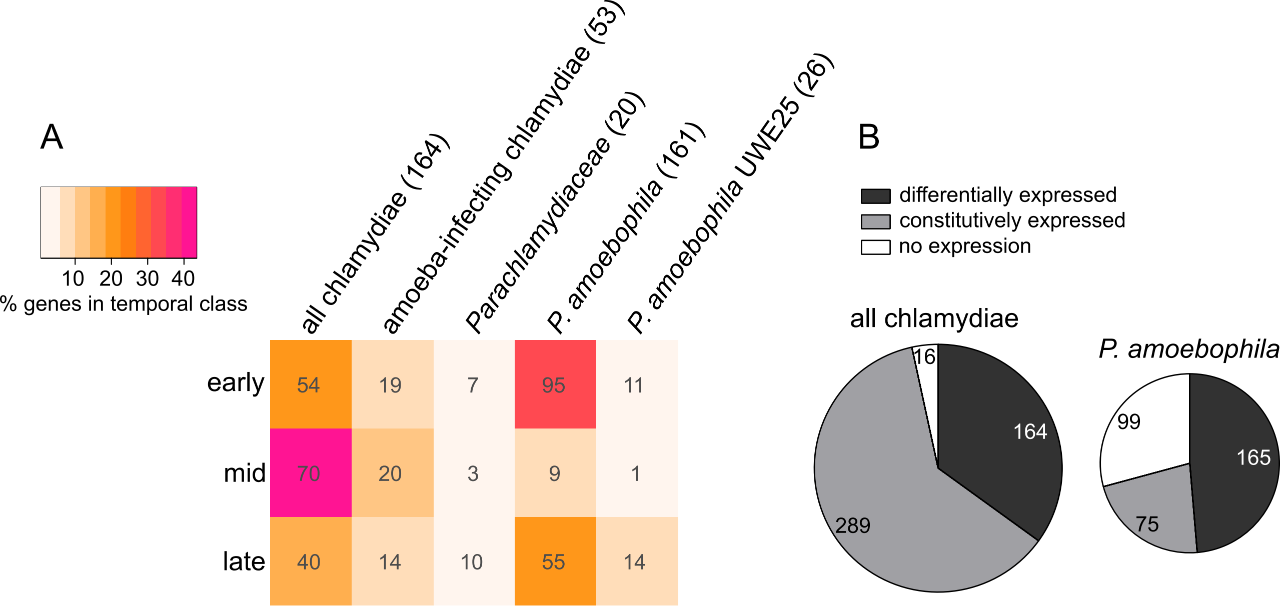
A
10 20 30 40
% genes in temporal class
all chlamydiae (164)
amoeba-infecting chlamydiae (53)
Parachlamydiaceae (20)
P. amoebophila (161)
P. amoebophila UWE25 (26)
early 54 19 7 95 11
mid 70 20 3 9 1
late 40 14 10 55 14
B
differentially expressed
constitutively expressed
no expression
all chlamydiae
16
164
289
P. amoebophila
99
165
75
