# Supplementary material for: Biphasic Metabolism and Host Interaction of a Chlamydial Symbiont
Source: mSystems. 2017 May 30;2(3):e00202-16. doi: 10.1128/mSystems.00202-16 (PMC5451489; doi:10.1128/mSystems.00202-16)
Supplement: TABLE S2 [file sys003172105st9.pdf]

Table S2. Overrepresented functions among *A. castellanii* genes

	GO term (ID)	Specifics on enriched genes (if applicable)	FDR
	**Upregulated genes**		
2 hpi	ATP binding (GO:0005524)	serine threonine kinases, ABC transporters	5.1E-06
	phosphorus-oxygen lyase activity (GO:0016849)	serine threonine kinases	3.6E-04
	cyclic nucleotide biosynthetic process (GO:0009190)	serine threonine kinases	3.6E-04
	mitochondrial respiratory chain complex III assembly (GO:0034551)		9.8E-04
	aminoacyl-tRNA ligase activity (GO:0004812)		1.6E-03
	tRNA aminoacylation for protein translation (GO:0006418)		1.6E-03
	GTPase activity (GO:0003924)	5x translation elongation and initiation, 5x small GTPases (signal transduction)	8.9E-03
	protein kinase activity (GO:0004672)	serine threonine kinases	2.2E-02
	ATPase activity, coupled to transmembrane movement of substances (GO:0042626)	ABC transporters	4.0E-02
	protein phosphorylation (GO:0006468)	serine threonine kinases	4.3E-02
	aminoacyl-tRNA editing activity (GO:0002161)		4.6E-02
	ATPase activity (GO:0016887)	8x ABC transporters, 2x ion/phospholipid pumps	4.8E-02
48 hpi	beta-galactosidase activity (GO:0004565)	beta-galactosidases	7.1E-04
	carbohydrate binding (GO:0030246)	mostly proteins involved in breakdown of (complex) sugars, e.g. alpha-mannosidases, beta-gal, cbm49 proteins	1.6E-03
	hexose metabolic process (GO:0019318)	mannosidases, fucosidases, galactosidases, PEP carboxykinases	2.3E-03
	phosphoenolpyruvate carboxykinase activity (GO:0004611)		2.1E-02
	phosphogluconate dehydrogenase (decarboxylating) activity (GO:0004616)		2.1E-02
	protein ADP-ribosylation (GO:0006471)		2.2E-02
	beta-N-acetylhexosaminidase activity (GO:0004563)		4.1E-02
	mannosidase activity (GO:0015923)		4.1E-02
96 hpi	phosphorus-oxygen lyase activity (GO:0016849)	serine threonine kinases	1.1E-25
	cyclic nucleotide biosynthetic process (GO:0009190)	serine threonine kinases	1.1E-25
	protein phosphorylation (GO:0006468)	serine threonine kinases and other protein kinases	6.0E-10
	phosphorelay sensor kinase activity (GO:0000155)	histidine kinases, two-component system	4.3E-08
	phosphorelay signal transduction system (GO:0000160)	histidine kinases, two-component system	6.8E-08
	ATP binding (GO:0005524)	mixed functions	5.2E-05
	heme binding (GO:0020037)	sterol/fatty acid biosynthesis	1.2E-02
	oxidoreductase activity, acting on paired donors, with incorporation or reduction of molecular oxygen (GO:0016705)	sterol/fatty acid biosynthesis	1.5E-02
	**Downregulated genes**		
2 hpi	no significant enrichment		
48 hpi	cyclic nucleotide biosynthetic process (GO:0009190)	serine threonine kinases	7.4E-11
	tRNA aminoacylation for protein translation (GO:0006418)		1.3E-09
	ribosome biogenesis (GO:0042254)		1.2E-08
	nucleocytoplasmic transport (GO:0006913)	Ras subfamily proteins (esp. Rab-GTPase) involved in signal transduction and protein transport (vesicle transport)	4.3E-06

	DNA replication initiation (GO:0006270)		1.1E-04
	mitochondrial respiratory chain complex III assembly (GO:0034551)		2.4E-03
	nucleosome assembly (GO:0006334)	histones and other assembly factors	2.4E-03
	cellular macromolecular complex assembly (GO:0034622)	nucleosome assembly, mitochondrial respiratory chain complex III assembly, tubulin alpha chain-like, spliceosomal tri-snRNP complex assembly	4.5E-03
	intracellular signal transduction (GO:0035556)	43x Ras-GTPases (esp. Rab, also Rac, Rap), 9x Rho-GTPases 38x serine threonine kinases, 5x histidine kinases, ARFs	5.4E-03
	translational elongation (GO:0006414)		1.2E-02
	regulation of cellular macromolecule biosynthetic process (GO:2000112)	transcription factors	1.4E-02
	intracellular protein transport (GO:0006886)	vesicular transport, fusion and movement proteins (28x Ras-like proteins of which 11 are Rab-proteins, SNARE protein, synthaxin-5)	1.8E-02
	RNA processing (GO:0006396)	proteins involved in tRNA modification/maturation (12x), ribosome assembly (8x), splicing/mRNA maturation (12x)	2.5E-02
	regulation of nucleobase-containing compound metabolic process (GO:0019219)	transcription factors	2.7E-02
	regulation of gene expression (GO:0010468)	transcription factors	3.1E-02
	protein ubiquitination (GO:0016567)	proteins with ubiquitin-protein transferase activity	4.5E-02
96 hpi	mitochondrial respiratory chain complex III assembly (GO:0034551)		4.3E-05
	protein heterodimerization activity (GO:0046982)	mixed functions	3.0E-04
	hydrolase activity, hydrolyzing N-glycosyl compounds (GO:0016799)	enzymes involved in base-excicion repair	8.0E-03
	base-excision repair (GO:0006284)		2.0E-02
	uracil DNA N-glycosylase activity (GO:0004844)	enzymes involved in base-excicion repair	3.6E-02
	nucleosome assembly (GO:0006334)	histones	3.6E-02
	protein ADP-ribosylation (GO:0006471)		3.7E-02
	NAD+ ADP-ribosyltransferase activity (GO:0003950)		4.1E-02
	DNA binding (GO:0003677)	histones, DNA replication factors, DNA repair factors, ssDNA binding	4.1E-02
